# Integrated Analysis of Survival, Physiological‐Biochemical, and Transcriptomic Changes Reveals the Impact of Saline Stress on the Freshwater Snail *Pomacea canaliculata*

**DOI:** 10.1002/ece3.71581

**Published:** 2025-07-03

**Authors:** Yingtong Chen, Fucheng Yao, Zhaoji Shi, Chunxia Zhang, Jimin Liu, Jiaen Zhang, Zhong Qin

**Affiliations:** ^1^ Guangdong Provincial Key Laboratory of Eco‐Circular Agriculture South China Agricultural University Guangzhou China; ^2^ College of Natural Resources and Environment South China Agricultural University Guangzhou China; ^3^ Guangdong Engineering Research Center for Modern Eco‐Agriculture and Circular Agriculture Guangzhou China; ^4^ Key Laboratory of Agro‐Environment in the Tropics Ministry of Agriculture and Rural Affairs Guangzhou China; ^5^ Department of Ecology, Institute of Hydrobiology Jinan University Guangzhou China

**Keywords:** *Pomacea canaliculata*, RNA‐seq, salinity acclimation, transcriptome

## Abstract

Salinity is an important abiotic stress that affects metabolic and physiological activities, breeding, development, and growth of mollusks. In this study, we investigated the effects of a range of water salinity on the apple snail *Pomacea canaliculata* , a highly invasive species and an important pest of rice. To examine the molecular response of *P. canaliculata* to salinity, we recorded young snails grown in a saline water environment for 4 months and compared their physiological and biochemical parameters with those of freshwater snails. We used RNA‐seq analysis to identify genes and biological processes involved in response to salinity. The results showed that saline water stress reduced the survival rate of the snail population, increased their feeding rate and snail weight, and led to an increase in shell strength and thickness, as well as a significant widening of the overall shell morphology. In female snails, the activities of CAT, SOD, and T‐AOC were significantly enhanced, while GSH activity, MDA content, and NOS activity showed significant decreases. In male snails, only MDA content exhibited a significant decrease, while ACHE activity showed a significant increase. Based on transcriptome analysis conducted for the liver and gills of the snails, a total of 1,569,678,584 raw reads were obtained from the nine libraries on the Illumina Novaseq 6000 platform. After preprocessing and the removal of low‐quality sequences, 1,560,932,792 clean reads were generated. The number of upregulated and downregulated differentially expressed genes (DEGs) in male snails after the saline stress was higher than that in female snails. The DEGs mainly involved oxidative stress, cellular regulation, and response. Saline concentration inhibited the hatching of eggs to a certain extent. Different levels of saline stress significantly affected the contents of free water, bound water, and enzyme activity of their eggs at different hatching stages. These findings provide theoretical support for understanding the saline tolerance of snails.

## Graphical abstract

In this study, we investigated the effects of a range of salinity on the apple snail *Pomacea canaliculata* , a highly invasive species and an important pest of rice. To examine the molecular response of *P. canaliculata* to salinity, we recorded young snails grown in a saline water environment for 4 months and compared their physiological and biochemical parameters with those of freshwater snails. These findings provide theoretical support for understanding the salt tolerance of snails.

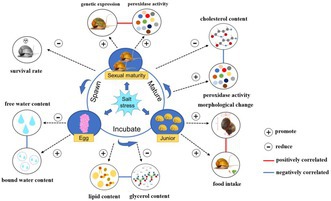

## 1 Introduction

*Pomacea canaliculata* is an extremely harmful aquatic invasive species that originated in the Amazon River basin of South America and has now spread to many countries and regions worldwide. It is considered to be one of the most malignant waterborne organisms (Yang et al. 2023; Zhou et al. 2016; Musri Musman et al. 2013). Due to its strong acclimation, broad diet, large food intake, high egg production, and fast reproduction rate, this invasive snail can quickly spread in environments such as rivers, lakes, and fields. It feeds on a variety of crops and aquatic plants, thus disrupting the food chain and posing a threat to the diversity of freshwater organisms and the functioning of the ecosystem in the invaded area (Ma et al. 2023; Corbin et al. 2022). In 2000, the apple snail was included in the list of the world's 100 worst invasive alien species (Manara et al. 2022). As an invasive species, *P. canaliculata* exhibits a remarkable ability to adapt to various environmental conditions. Investigating its environmental adaptability is crucial for developing targeted ecological management strategies to mitigate its detrimental impacts on native species and the overall ecosystem.

Salinity is one of the most important environmental factors in aquatic environment, which greatly affects the survival, reproduciton, development, growth, metabolism, immune function, and physiological functions of mollusks (Martyniuk et al. 2022; Koudenoukpo et al. 2021; Gaiser et al. 2006). *P. canaliculata* is classified as an osmoconforming mollusk, and its population's ability to acclimate to saline stress indicates a potential for invading estuarine habitats (Koudenoukpo et al. 2021). *P. canaliculata* can potentially survive in environments with salinity levels of ≤ 5.0 ppt, with survival rates varying by age class based on shell height, following the order: older > mature > juvenile snails (Liu, Liu, et al. 2022). Qin et al. found that *P. canaliculata* can survive for at least 5 days at a salinity of 12.0 ppt, over 30 days in environments with salinity ranging from 0 to 6 ppt, and can maintain normal physiological activities under salinity conditions of 0 to 4 ppt. As environmental salinity gradually increases, *P. canaliculata* exhibits enhanced saline tolerance; however, higher salinity levels lead to significant decreases in average daily weight gain, specific growth rate, and egg hatching rate (Qin et al. 2022, 2020). Experimental results confirmed that in low‐salinity environments, *P. canaliculata* can absorb more abundant inorganic ions, thereby promoting its growth, and it is speculated that this may further improve its salinity tolerance (Yang et al. 2016). However, the tolerance and long‐term acclimation mechanisms of freshwater and saline water environmental stress on *P. canaliculata* have not been studied at the transcriptome level to identify the genes responsible for salinity regulation, which affects the understanding of the fundamental mechanism underlying acclimation to fluctuations in salinity. When the environmental salinity changes abnormally, it causes a variety of physiological stress reactions in *P. canaliculata*, and the production of reactive oxygen species (ROS) increases accordingly (Polyak et al. 2022; Batista et al. 2023). Owing to variation in the salinity of the aquatic environment, *P. canaliculata* have evolved various physiological strategies for salinity acclimation. In the coastal regions of southern China, the apple snails have spread to the coastal brackish water wetland ecosystem, and have been found in habitats such as fish ponds, tidal flats, and farmland where both fresh and saline water are present, including the mangrove wetlands in the coastal areas of South China, with the presence of *P. canaliculata* and their egg masses (Liu, Liu, et al. 2022).

Enzymes and transporters play a crucial role in maintaining internal osmotic and ionic homeostasis in response to fluctuating water salinity, thus actively participating in salinity acclimation and osmoregulation (Andreeva et al. 2020). The identification of candidate genes associated with salinity changes is of paramount importance in elucidating the molecular foundation underlying this essential physiological process (Ren et al. 2020). The transcriptome represents a collection of genes that exhibit dynamic expression patterns, which are contingent upon the physiological state of organisms and are responsive to external environmental factors (Andreeva et al. 2020; Price et al. 2007). These investigations have allowed the elucidation of several differentially expressed genes (DEGs) and pathways associated with changes in water salinity.

With the rapid development of molecular technologies, it is possible to study the ecological and physiological mechanisms of *P. canaliculata* using gene expression analysis. Prior studies on *P. canaliculata* have primarily focused on examining the effects of salinity on various aspects such as survival, larval and juvenile development, oxygen consumption, ammonia excretion, growth, and energy budget (Qin et al. 2022; Yang et al. 2018). Although basic data such as morphological traits and survival rates may seem sufficient to address the issue, they only describe the phenotypic outcomes resulting from environmental stress and fail to uncover the molecular mechanisms driving physiological acclimation. *P. canaliculata’*s survival under saline water conditions is primarily regulated by gene expression‐mediated phenotypic changes rather than immediate morphological alterations. Analyzing DEGs allows precise identification of stress‐related pathways, including osmotic regulation, ion transport, energy metabolism, and antioxidant defense, which are vital for long‐term acclimation processes not directly observable through morphology or survival data alone.

Importantly, transcriptomic data capture early molecular responses to stress before visible phenotypic changes occur, providing crucial insights for predicting long‐term survival and invasion potential. Many invasive species rely on gene‐regulated phenotypic plasticity to colonize new environments. Insufficient knowledge is available concerning the molecular pathways associated with acclimatory mechanisms in response to salinity changes in *P. canaliculata*. Consequently, there is a compelling need to investigate the salinity acclimation of *P. canaliculata* at the transcriptional level to unravel the underlying fundamental mechanisms involved in water salinity acclimation and it remains unclear whether this invasive species can establish stable populations in coastal wetlands.

In this study, we aimed to investigate the survival mechanisms of *P. canaliculata* under saline stress. To this end, we conducted a 4‐month saline stress experiment. The specific objectives were to: (1) evaluate the effects of saline stress on survival rate, food intake, body weight, morphological characteristics, and egg mass production; and (2) compare gene expression profiles of two major osmoregulatory organs, the gills and liver, under control (0 ppt) and saline stress (2 ppt) conditions using RNA sequencing (RNA‐seq).

## 2 Materials and Methods

### 2.1 Experimental Snails and Salinity Stress Treatment

*Pomacea canaliculata* snails were reared in open‐air cement ponds located at the Ecological Teaching and Research Farm (113°1′E, 23°9′N) of South China Agricultural University (SCAU) in Guangzhou, China. *P. canaliculata* individuals were collected from the wild field when their shell height reached approximately 10 mm (±2 mm) and were subsequently brought into the laboratory for cultivation. The snails were cultivated indoors at a temperature of 26°C ± 1°C for 10 days and placed in plastic aquaria (45 cm ×35 cm ×35 cm) at a depth of about 20 cm. Each day, excess food was provided, and water was timely changed. The lettuce from the previous day was removed and replaced with fresh lettuce to maintain a state of excess food.

Snails with normal appearance and strong vitality were selected for the experiment. The aquariums were covered with mesh to prevent the snails from escaping. The snails were reared under natural lighting conditions. Every 3 days, two‐thirds of the water was pumped out, and aerated tap water containing saturated calcium carbonate was added to a depth of 15 cm. Observations were made during the first 10 days of the experiment to determine a feeding amount of 100 g per aquarium (with surplus food provided daily).

A total of 80 snails were divided into each aquarium containing aerated water. The experiment consisted of two treatments: one had saline concentration adjusted to 2 ppt (our previous salinity measurements in coastal wetlands inhabited by *P. canaliculata* indicated an average salinity of 2 ppt) using commercially available marine salt mixtures (contain 98% NaCl along with trace minerals including MgSO_4_, CaSO_4_ and K_2_SO_4_, was produced under the product standard number Q/320482YDWL003) and the other without saline treatment, each treatment replicated four times. The survival mechanism of *P. canaliculata* was determined by assessing its response when the operculum was pried open. The survival rate in each aquarium was recorded every 5 days, and the daily food intake and snail weight were recorded every 10 days. Any dead snails were promptly removed. The duration of continuous exposure for adult snails was 80 days. The hatched *P. canaliculata* snails were not further exposed to saline conditions; they were placed in tap water for observation and data collection.

### 2.2 Shell Strength and Shell Thickness Measurement

In both the saline water‐treated group and the control group, 5 male and 5 female snails with similar body sizes were randomly selected from the aquariums. Their shell thickness and shell compressive strength (shell strength) were measured. The shells and flesh were carefully separated without damaging the snail shells using dissecting scissors and a surgical knife. The shell compressive strength was measured using an Edgewood spring pressure tester (model HP‐200) equipped with a digital force gauge (accuracy: 0.1 N). The snail was placed on the loading platform with the shell opening facing downward, and the force gauge was used to compress the shell until it was crushed. The maximum force recorded during the shell‐crushing process represented the shell compressive strength. The shell thickness was measured at three randomly selected locations around the damaged area using a spiral micrometer (model DL321025B) with an accuracy of 0.001 mm. The average of these measurements provided an estimation of the shell thickness.

### 2.3 Morphological Characteristics Measurement

**FIGURE 1:**
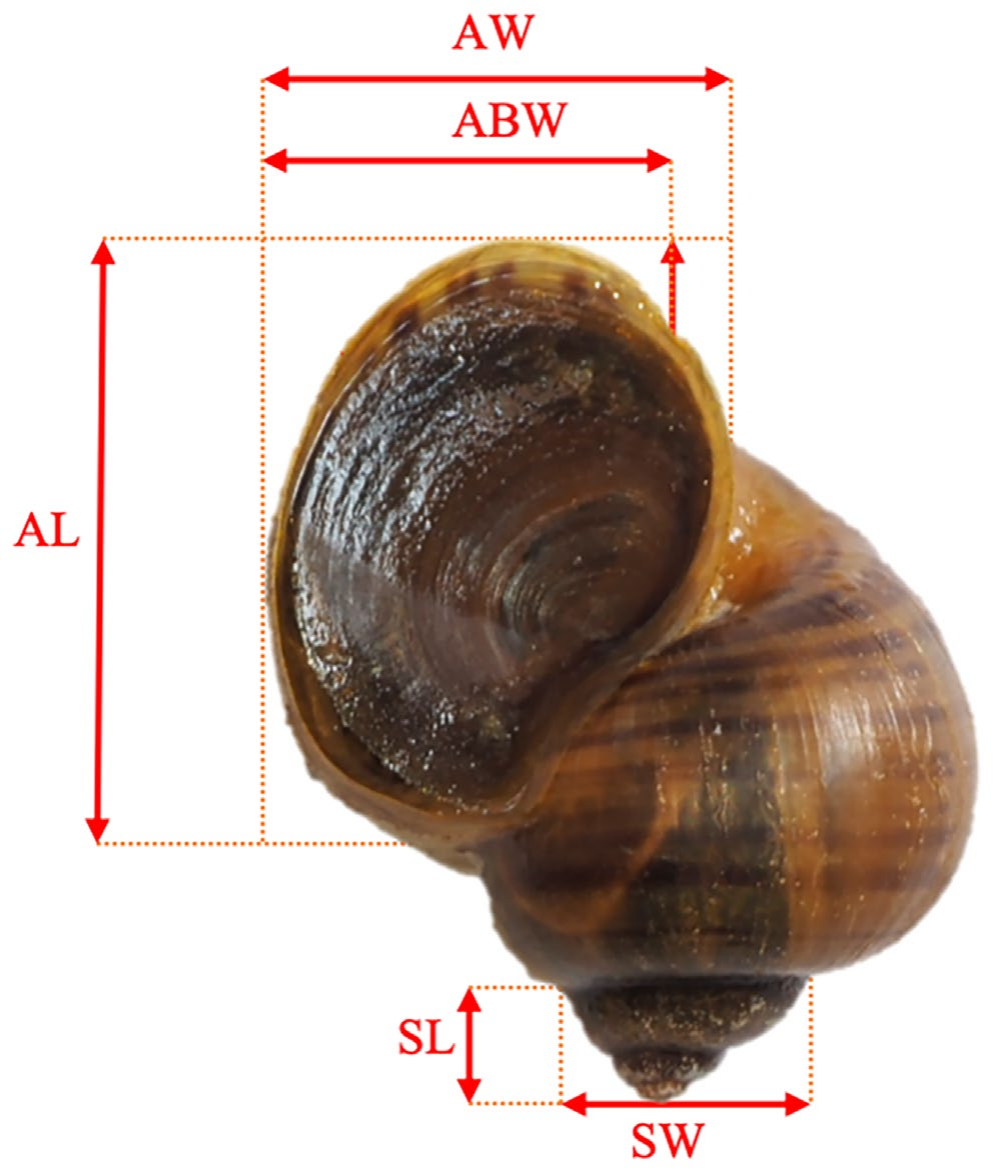
Morphological indicators of *P. canaliculata*.

Morphological measurements of the snail shells were conducted using intact individuals of the selected snail species. The specimens were fixed and two‐dimensional images were captured using a camera. The software Image‐Pro Plus 6.0 was employed to measure the morphological parameters of the snail shells. The following measurements were selected: spire length (SL), spire width (SW) (Swinehart et al. 1998), height from all layers (HL), inner lip length (IL), basal lip height (BL), aperture length (AL), aperture basal width from the lowermost part of the aperture to the periphery (ABW), aperture inner length (AL’), and aperture width (AW). The measurement sites and parameters are illustrated in the Figure 1. To account for the potential influence of individual size differences on the analysis of morphological features, data correction was performed using shell height (HL), aperture length (AL), and aperture width (AW) as covariates for all measured parameters.

### 2.4 Saline Stress Treatment of Egg Masses

Egg masses of *P. canaliculata* were collected from a natural population on September 22, during the peak oviposition season in summer. Intact egg masses with visible surface mucus and bright red coloration, which indicated they were freshly laid on the same day, and randomly selected using a tool to assist with detachment. The egg mass collection was conducted separately from the juvenile snail experiments, which took place at a different time. Each egg mass was placed into its respective culture dish, with gauze netting stretched across the top to support the egg mass. Upon hatching, the juveniles passed through the mesh into the dish, where they were incubated and subsequently counted. Solutions of 0, 2, and 5 ppt salinity were prepared using commercially available sea saline crystals (same contents for the adult survival treatments) and added into the corresponding spray bottles. The CK treatment involved no liquid application, simulating the natural hatching conditions of *P. canaliculata* eggs in the environment. The egg masses were wetted twice daily, at 10:00 AM and 10:00 PM respectively, ensuring the surface was just covered with water each time. The hatched snails were transferred promptly to prevent their escape.

Using the CK group as a reference, an inhibition rate (IR) greater than 0 indicates that the treatment exerts an inhibitory effect on egg mass hatching, whereas an IR less than 0 suggests that the treatment promotes egg mass hatching. The hatching rate (HR, %) and inhibition rate (IR, %) of *P. canaliculata* egg masses were calculated using the following formulas:

$$\mathrm{HR}=\frac{\mathrm{THS}}{\mathrm{TNE}}\times 100\%$$

$$\mathrm{IR}=\frac{\mathrm{HR}\left(\mathrm{CK}\right)-\mathrm{HR}\left(\text{TREATMENT}\right)}{\mathrm{HR}\left(\mathrm{CK}\right)}\times 100\%$$

In the formulas, THS represents the total number of hatched snails, TNE denotes the total number of eggs, HR(CK) refers to the hatching rate of the control group, and HR(TREATMENT) indicates the hatching rate of the treatment group. The treatment period for egg masses was 30 days.

### 2.5 Enzyme Activity Measurement

The specific tissues were dissected and promptly frozen in liquid nitrogen for preservation at −80°C in a freezer. During the measurements, a mass‐volume ratio of 1:9 (tissue to pre‐chilled physiological saline) was prepared. The tissue was homogenized in an ice‐water bath using an electric high‐speed tissue homogenizer to obtain a 10% tissue homogenate. Subsequently, the homogenate was centrifuged at 3000 rpm for 10 min at 4°C, and the resulting supernatant was collected for the determination of activities of CAT, GSH, ACHE, NOS, SOD, T‐AOC and MDA content (Catalan et al. 2006)

### 2.6 Statistical Analyses

Statistical analysis was conducted using one‐way analysis of variance (ANOVA) followed by least significant difference (LSD) or Games–Howell post hoc tests for multiple comparisons on survival rate, food intake, weight, shell strength and shell thickness, morphological changes, and enzyme activity of snails. IBM SPSS Statistics 26 software was used for the analysis, and graphs were created using Origin 8.0. Significance was set at *p* < 0.05, or *p* < 0.01 (Table 1).

**TABLE 1:** Statistical methods used for data analysis.

Variable	Statistical test	Post hoc test	Significance level
Survival rate	One‐way ANOVA	Games–Howell	*p* < 0.05, or *p* < 0.01
Food intake	One‐way ANOVA	Games–Howell	*p* < 0.05, or *p* < 0.01
Weight	One‐way ANOVA	Games–Howell	*p* < 0.05, or *p* < 0.01
Shell strength	One‐way ANOVA	LSD	*p* < 0.05, or *p* < 0.01
Shell thickness	One‐way ANOVA	LSD	*p* < 0.05, or *p* < 0.01
Morphological changes	One‐way ANOVA	LSD	*p* < 0.05, or *p* < 0.01
Enzyme activity	One‐way ANOVA	LSD	*p* < 0.05, or *P* < 0.01
Hatchability and inhibition rate	One‐way ANOVA	LSD	*p* < 0.05
Physiological and biochemical substance content	One‐way ANOVA	LSD	*p* < 0.05
	One‐way ANOVA	LSD	*p* < 0.05
Antioxidative System Indices	One‐way ANOVA	LSD	*p* < 0.05

### 2.7 Total RNA Extraction, Library Construction and Illumina Sequencing

The integrity, concentration, and purity of the RNA samples were assessed using agarose gel electrophoresis and Nanodrop analysis. To construct a strand‐specific library, a method for removing ribosomal RNA (rRNA) was employed. Firstly, total RNA was subjected to rRNA depletion. Subsequently, the RNA was fragmented into short fragments of 250–300 bp. The fragmented RNA served as a template for the synthesis of the first cDNA strand using random oligonucleotides as primers. Then, the RNA template was degraded using ribonuclease H, and the second cDNA strand was synthesized using DNA polymerase I and four deoxyribonucleotide triphosphates. The purified double‐stranded cDNA was subjected to end repair, A‐tailing, and ligation with sequencing acclimator. The cDNA library was size‐selected using AMPure XP beads to obtain fragments of approximately 350–400 bp. The second strand of cDNA, which contained uracil, was selectively degraded using uracil‐specific excision reagents. Finally, PCR amplification was performed to generate the library. If the library exhibited an insert fragment length distribution of approximately 250–300 bp and an effective concentration greater than 2 nM, it was subjected to paired‐end 150 bp sequencing using the Illumina platform.

### 2.8 Analysis of Differentially Expressed Protein Codes

The raw data were processed using Fastp to remove acclimator sequences and low‐quality sequences, resulting in clean data with a minimum read length of 75 bp. The genome sequence and annotation file of snail were obtained from the NCBI database (http://www.ncbi.nlm.nih.gov/protein/). The clean data was aligned to the reference genome using Hisat2 with default parameters. Stringtie software was then used to assemble and identify novel transcripts based on the genome‐aligned results.

Using the merged transcripts generated by Stringtie as the reference, gene‐level quantification was performed, and a read count matrix was generated. The read count matrix was imported into R 3.6.3 software, and the R package edgeR was used for differential expression analysis of protein‐coding genes. Genes with a fold change greater than 1 (|log2(Fold change)| > 1) and a false discovery rate (FDR) less than 0.05 were considered as differentially expressed genes between groups. Additionally, Tbtools was used for gene ontology (GO) and Kyoto Encyclopedia of Genes and Genomes (KEGG) pathway enrichment analysis of the differentially expressed genes (Table 2).

**TABLE 2:** Water salinity and control treatments for the snails and their eggs.

Information on the salinity treatments	Experimental treatments for snails	Experimental egg masses
CK	0 ppt	Untreated
0 ppt	N/A	0 ppt
2 ppt	2 ppt	2 ppt
5 ppt	N/A	5 ppt

*Note:* ‘N/A’ (not applicable) is used when a specific treatment is not applicable. “Untreated” refers to experimental subjects that have not undergone any treatment. All artificial water used in this study was aerated tap water.

## 3 Results

### 3.1 Survival

**FIGURE 2:**
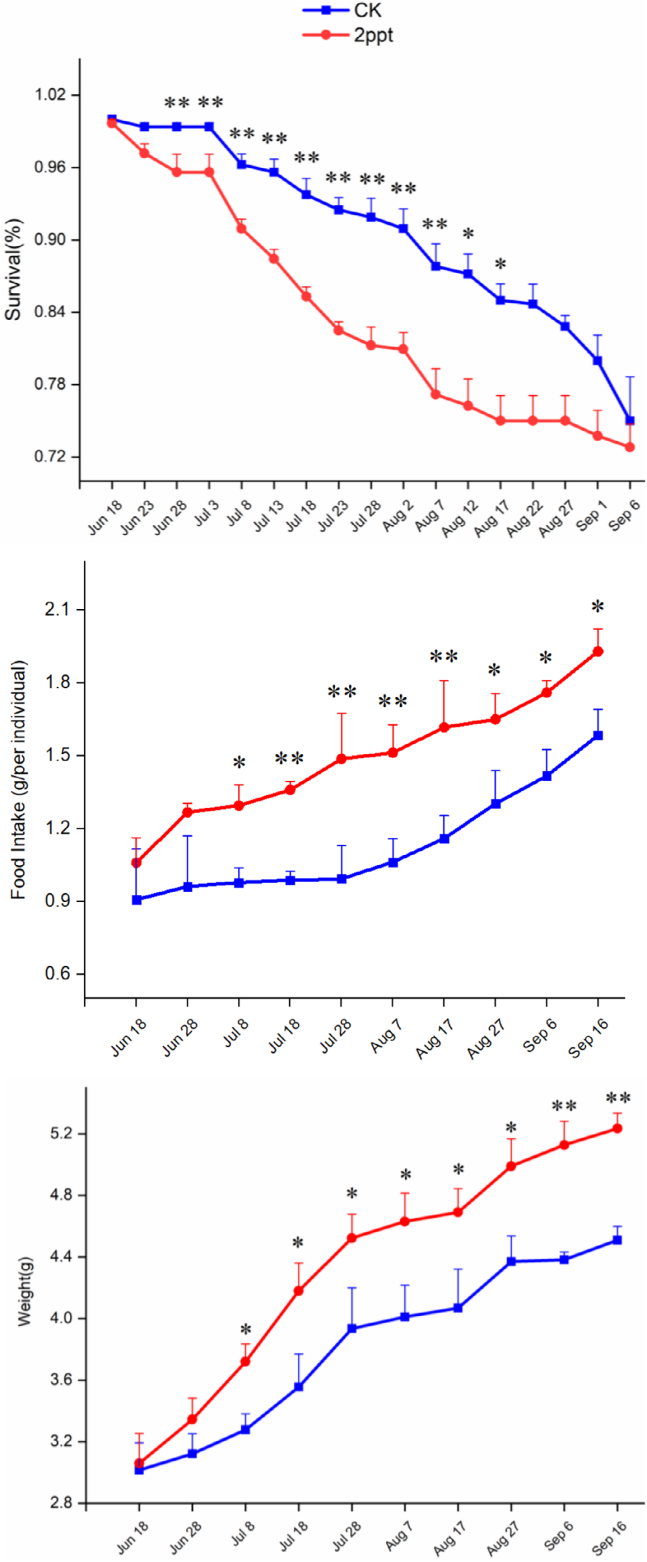
Survival, food intake, and weight of *P. canaliculata* in different saline treatments. CK represents the treatment that snails were kept in a freshwater environment without salinity. The error bars represent the standard error (SE). The feeding amount and snail weight values in the figure represent the average feeding amount and body weight per individual. A single asterisk (*) indicates *p* < 0.05, while double asterisks (**) indicate *p* < 0.01.

Compared with the control group (0 ppt), the survival rate of *P. canaliculata* declined in a 2 ppt water salinity environment on July 8th (Figure 2). The survival rates of *P. canaliculata* in the control and 2 ppt salinity treatment were 99% and 91%, respectively, showing a significant difference at a highly significant level (*p* < 0.01). After July 8th, the survival rates of the 2 ppt salinity treatment group were significantly lower than the control until August 17th. Subsequently, with increasing treatment time, the difference in survival rates between the 2 ppt treatment group and the control group gradually diminished. At the end of the experiment, the survival rates in the control and 2ppt treatment groups were 75% and 73%, respectively, with no significant difference (*p* > 0.05). The results of repeated measures analysis of variance indicated a highly significant effect of treatment time on the survival rate of *P. canaliculata* (*F*_2,16_ = 102.992; *p* < 0.001), and a significant interaction between the water salinity stress and the treatment time (*F*_2,16_ = 4.481; *p* < 0.05).

### 3.2 Food Consumption

In both treatments, *P. canaliculata* exhibited normal eating activity at the beginning of the treatments. While from July 8th, the snails in the saline environment showed significantly higher food intake compared with the control (*p* < 0.05), with daily average eating amounts of 1.30 and 0.98 g for the saline water and control treatments, respectively. Throughout the experiment, the snails in the saline treatment consistently demonstrated significantly higher food intake than the control (*p* < 0.05), with the greatest difference observed on July 28th, where the food intake in the saline environment was 1.50 times higher than that of the control group. The results of the repeated measures analysis of variance indicated a highly significant effect of the treatment time on food intake (*F*_2,9_ = 5.197; *p* < 0.05), and a significant interaction between the water salinity stress and the treatment time (*F*_2,9_ = 3.233; *p* < 0.05).

### 3.3 Weight

Overall, the population weight of the snails in both treatments showed a gradual increase, although there were some periods during which the average weight gain slightly reduced due to the occurrence of mortality among larger individuals. After June 8th, the snail weight in the saline water treatment was significantly higher than the CK, with average weights of 3.72 and 3.28 g, respectively. In the later stage of the experiment, the snail population in the saline treatment consistently exhibited significantly higher weight than the CK, with a highly significant difference observed between September 6th and September 16th. The body weight growth rate exhibited a pattern of initial increase followed by a subsequent decline. In the CK group, the highest growth rate (19%) was observed on August 17th, whereas in the 2 ppt treatment group, the peak (15%) occurred on July 15th, which was approximately the midpoint of the experiment. By the end of the experiment, the average body weight in the CK had increased from 3.015 to 4.51 g per individual, while in the 2 ppt group, it increased from 3.66 to 5.235 g per individual. The snail weight in the saline treatment was 1.17 and 1.16 times higher than the corresponding CK. The results of the repeated measures analysis of variance indicated a highly significant effect of the treatment time on weight (*F*_2,9_ = 60.084; *p* < 0.001), while the interaction between the salinity stress and the treatment time was not significant (*F*_2,9_ = 2.153; *p* > 0.05) (Figure 2).

### 3.4 Shell Strength and Thickness

**FIGURE 3:**
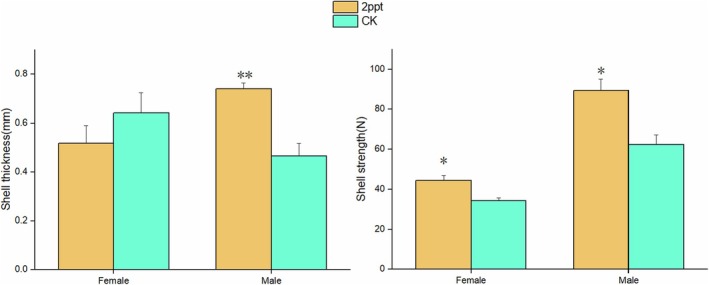
Shell thickness and shell strength of *P. canaliculata* in saline treatments. CK represents the treatment that snails were kept in a freshwater environment without salinity. The error bars represent the standard error. The data in the figure represent the average shell thickness and shell strength per individual. A single asterisk (*) indicates *p* < 0.05, while double asterisks (**) indicate *p* < 0.01.

Under saline stress treatment, the shell thickness of male *P. canaliculata* significantly increased (*p* < 0.01), with the saline treated snails having a shell thickness approximately 1.6 times greater than the control. The response of shell thickness to saline treatment varied slightly between male and female *P. canaliculata*. In the control, the shell thickness of males was lower than that of females, while the opposite trend was observed under saline treatment. Both snail sex and saline treatment had a noticeable impact on shell strength in *P. canaliculata*, with males exhibiting greater shell strength than females in both saline stress and freshwater treatments. After saline treatment, both male and female snails showed a significant enhancement in shell strength (*p* < 0.05), particularly in males, where the saline treatment group had a snail shell strength 1.4 times greater than the control group (Figure 3).

### 3.5 Morphological Changes

**FIGURE 4:**
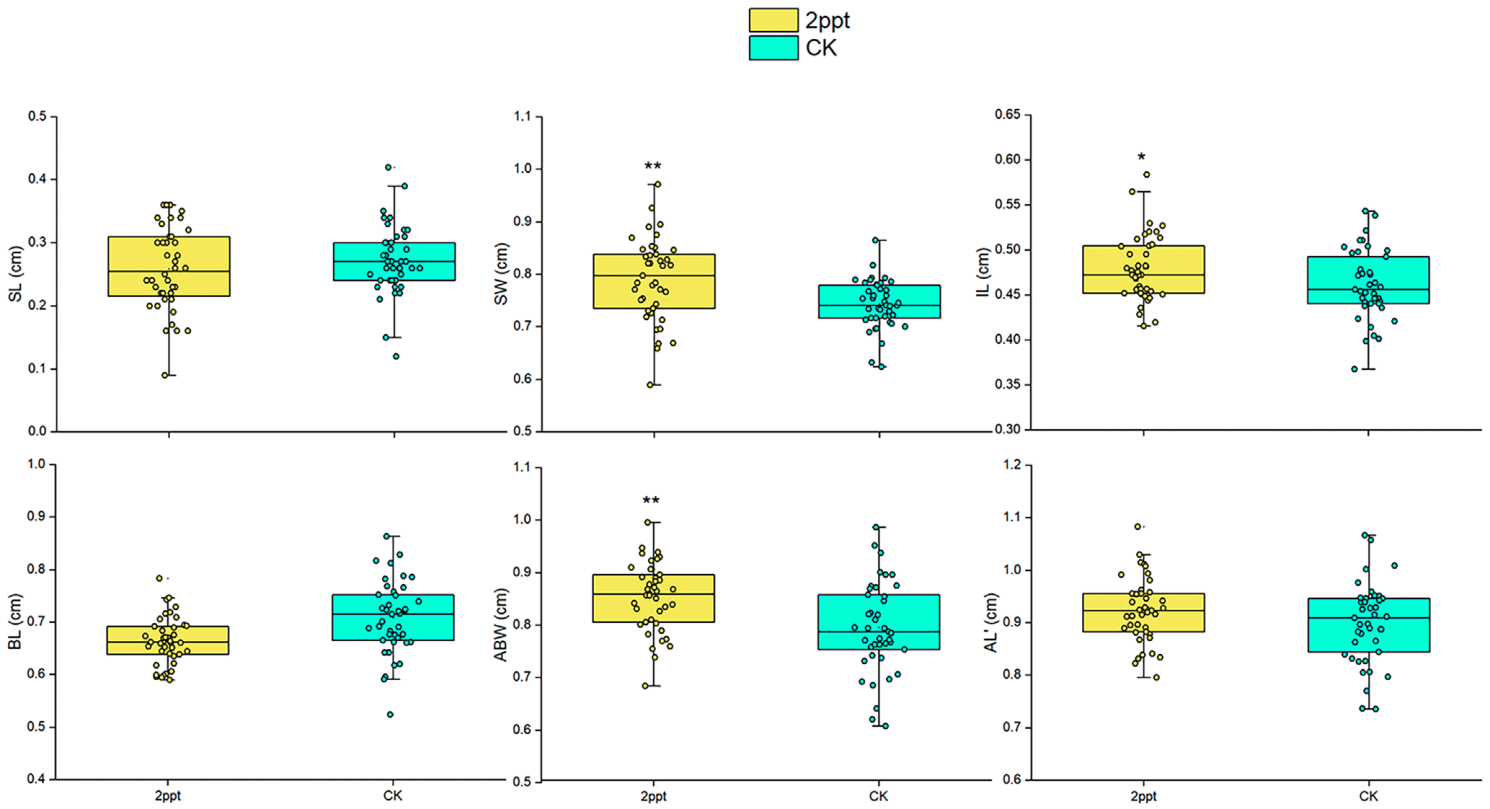
Morphological changes of *P. canaliculata* in different saline treatments. CK represents the treatment that snails were kept in a freshwater environment without salinity. The error bars represent the standard error. A single asterisk (*) indicates *p* < 0.05, while double asterisks (**) indicate *p* < 0.01.

For morphological parameters of *P. canaliculata,* the results showed no significant difference in spire length (SL) between the two groups (*p* > 0.05) after correcting for body whorl height (HL) as a covariate. However, there was a significant difference in spire width between the two groups, with values of 0.79 and 0.74 cm for the long‐term saline treated snails and the control, respectively (*p* < 0.01). When aperture length (AL) was used as a covariate, the inner lip length (IL) of snails in the saline treatment was 0.48 cm, significantly longer than 0.46 cm of the control group (*p* < 0.05). The difference in basal lip (BL) height between the two groups was not statistically significant (*p* > 0.05). After correcting for aperture width (AW), the aperture basal width (ABW) exhibited mean values of 0.85 cm, while only 0.80 cm in the control group (*p* < 0.01). The aperture inner lengths (AL’) between the two groups were similar and showed no significant difference (*p* > 0.05) (Figure 4).

### 3.6 Enzyme Activity

**FIGURE 5:**
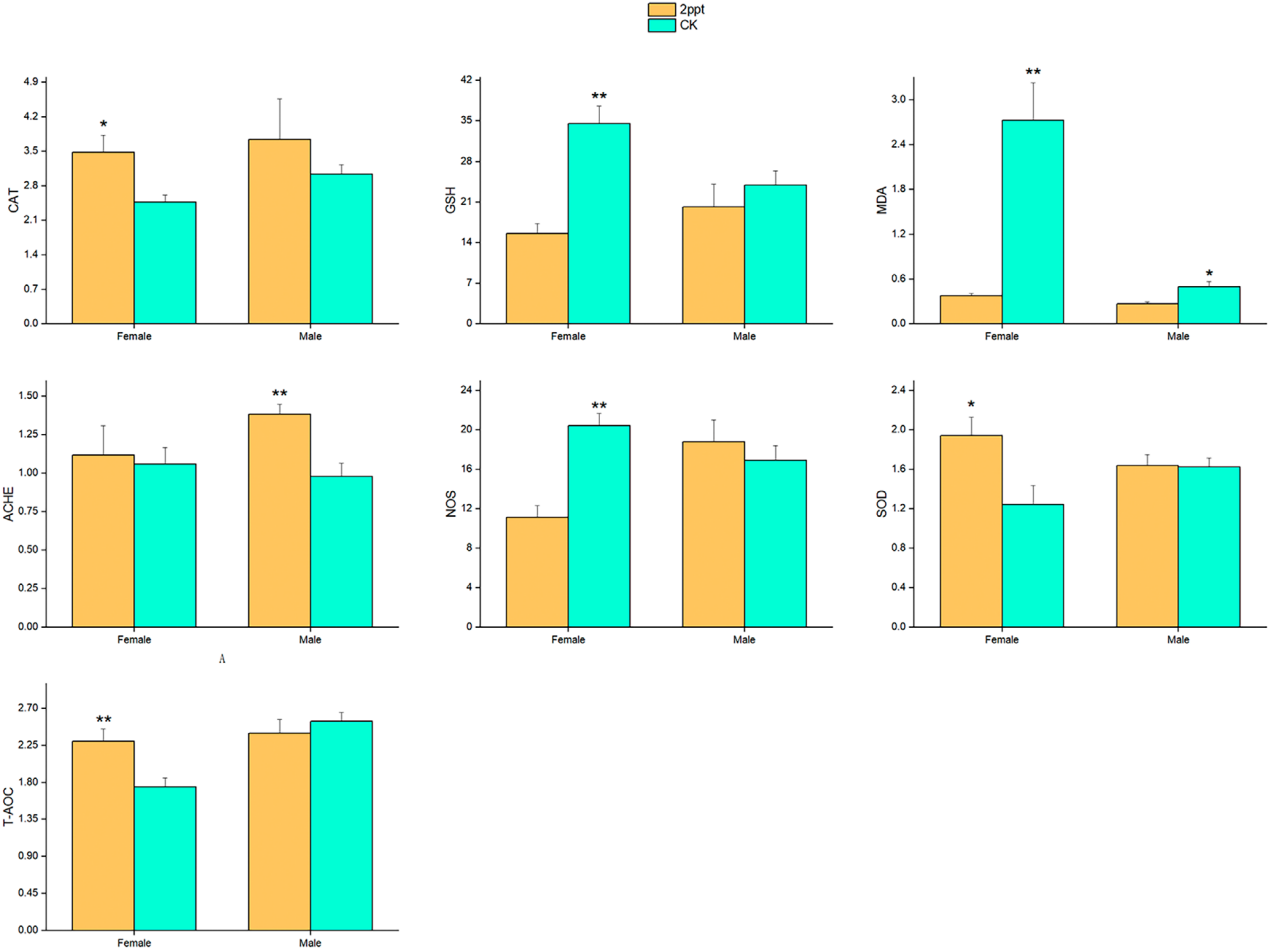
Enzyme activity of *P. canaliculata* in different saline treatments. CK represents the treatment that snails were kept in a freshwater environment without salinity. The error bars represent the standard error. All antioxidant system indicators were normalized based on protein content. A single asterisk (*) indicates *p* < 0.05, while double asterisks (**) indicate *p* < 0.01. All enzyme activities were normalized to protein content. The units for CAT, ACHE, NOS, SOD, and T‐AOC are expressed as U/mg protein; GSH is expressed as μmol/g tissue, and MDA as nmol/g tissue.

In the long‐term saline water environment, the activities of CAT, SOD, and T‐AOC in female snails significantly enhanced, while GSH, MDA, and NOS acitvities significantly decreased. In male snails, only MDA content exhibited a significant decrease, and ACHE activity showed a significant increase (*p* < 0.05). There existed a significant interaction between salt treatment and sex for GSH (*F*_1,8_ = 7.272, *p* < 0.05), MDA (*F*_1,8_ = 17.191, *p* < 0.01), NOS (*F*_1,8_ = 14.657, *p* < 0.01), and T‐AOC (*F*_1,8_ = 7.963, *p* < 0.05) (Figure 5).

### 3.7 Transcriptome Sequencing, De Novo Assembly and Alignment

High‐throughput sequencing was used to systematically analyze gene expressions in livers and gills with or without saline treatment. We performed deep sequencing of RNA samples from snail gills and livers. In total, 1,569,678,584 raw reads (150 bp) were obtained from the nine libraries on the Illumina Novaseq 6000 platform. After preprocessing and removal of low‐quality sequences, 1,560,932,792 clean reads were generated. Additionally, the proportion of Q30 bases exceeded 95%, which is essential for ensuring accurate sequencing (Table 3). The clean reads were mapped to the *P. canaliculata* reference genome. In all, the uniquely mapping ratios for the 79.4% and 80.4% were obtained from saline‐treated liver tissue of female and male snails, 80.3% and 79.6% for saline‐treated gill tissue of female and male snails, 79.0% and 78.4% control liver tissue of female and male snails, and 81.3% and 81.5% for control gills of female and male snails, respectively, indicating high levels of gene expression in both groups.

**TABLE 3:** Summary of trimming and reads results of the sequences generated from livers and gills of the *P. canaliculata* with or without the saline treatments.

Sample	Raw reads	Clean reads	Q30 (%)	GC content (%)
SFL1	4,51,56,894	4,49,25,618	95.24	49.09
SFL2	5,12,44,282	5,10,04,706	95.63	51.4
SFL3	5,07,67,440	5,04,74,208	95.66	51.75
SFL4	5,70,31,684	5,67,22,334	95.63	51.4
SML1	4,74,91,730	4,72,17,796	95.13	49.11
SML2	4,78,35,368	4,75,52,158	95.54	51.57
SML3	4,53,24,356	4,49,16,414	95.83	51.15
SML4	4,47,39,836	4,45,10,574	95.52	51.08
SFG1	4,81,80,600	4,79,24,068	95.46	45
SFG2	4,82,87,456	4,80,60,452	95.47	44.43
SFG3	4,89,89,448	4,87,56,954	95.18	43.85
SFG4	5,44,25,312	5,41,47,474	95.55	44.71
SMG1	4,24,06,540	4,21,86,766	95.09	43.38
SMG2	4,40,50,950	4,38,43,060	95.49	43.38
SMG3	4,29,52,140	4,27,21,446	95.4	44.3
SMG4	4,16,63,690	4,14,48,540	95	43.2
CFL1	4,39,73,042	4,35,98,566	95.61	50.95
CFL2	4,35,61,302	4,32,45,780	95.54	49.18
CFL3	4,72,80,110	4,69,19,564	95.57	49.63
CFL4	5,79,74,724	5,76,44,298	95.84	51.06
CML1	4,30,61,962	4,27,86,376	95.64	49.82
CML2	6,16,53,842	6,12,94,066	95.62	48.51
CML3	5,21,53,760	5,18,53,138	95.43	51.34
CML4	5,66,24,968	5,63,26,906	95.63	48.55
CFG1	5,53,52,954	5,50,82,678	95.25	44.38
CFG2	4,48,41,194	4,46,21,496	95.56	44.29
CFG3	4,85,34,750	4,83,12,988	95.42	44.48
CFG4	4,14,86,286	4,12,98,174	95.11	43.66
CMG1	5,86,09,936	5,83,27,806	95.47	45.29
CMG2	5,57,16,670	5,54,48,608	95.43	45.06
CMG3	4,83,92,476	4,81,32,426	95.5	45.45
CMG4	4,99,12,882	4,96,27,354	95.12	45.2
Total	1,56,96,78,584	1,56,09,32,792		

*Note:* SFL represents saline‐treated liver tissue of female snails; SML represents saline‐treated liver tissue of male snails; SFG represents saline‐treated gills tissue of female snails; SMG represents saline‐treated gills tissue of male snails; CFL represents without saline‐treated liver tissue of female snails; CML represents without saline‐treated liver tissue of male snails; CFG represents without saline‐treated gills tissue of female snails; CMG represents without saline‐treated gills tissue of male snails. Four replicates of each treatment were carried out in RNA‐seq analysis.

### 3.8 Identification and Analysis of Differentially Expressed Genes

**FIGURE 6:**
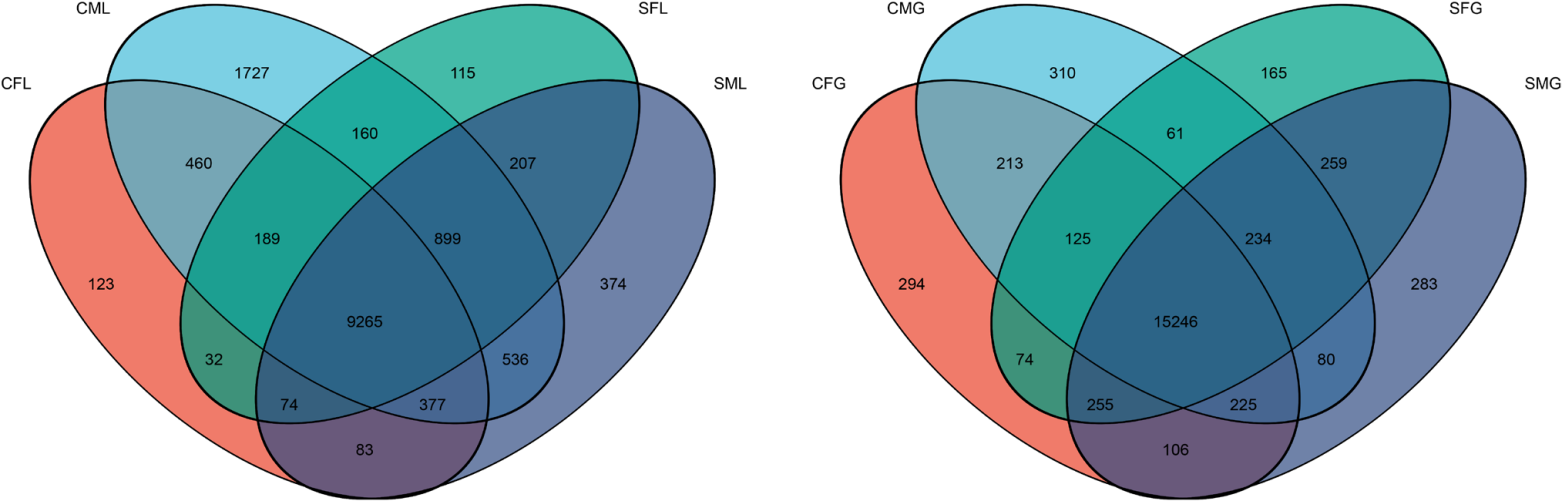
The different colored circles represent the transcripts of DEGs in a sample based on expression screening, and the values represent the number of DEGs to different samples. SFL represents saline‐treated liver tissue of female snails; SML represents saline‐treated liver tissue of male snails; SFG represents saline‐treated gills tissue of female snails; SMG represents saline‐treated gills tissue of male snails; CFL represents without saline‐treated liver tissue of female snails; CML represents without saline‐treated liver tissue of male snails; CFG represents without saline‐treated gills tissue of female snails; CMG represents without saline‐treated gills tissue of male snails. Four replicates of each treatment were carried out in RNA‐seq analysis.

To explore the potential response of *P. canaliculata* to salinity stress, we conducted transcriptomic analysis on liver and gill tissues collected from the endpoint of the study. A Venn diagram was employed to identify the commonly DEGs across the libraries. We focused on the analysis of DEGs that exhibited transcriptional regulation at specific time points. Within the livers and gills, a total of 9265 upregulated DEGs and 15,248 downregulated DEGs were identified, representing the shared gene expression changes in response to the experimental conditions (Figure 6).

### 3.9 Functional Annotation and Gene Ontology Classification

To analyze the transcriptome profile of *P. canaliculata* and its gene models, we first filtered reads from two tissues and mapped them separately to the *P. canaliculata* reference genome. Functional annotation gave information on the transcripts, and genes were aligned with public protein databases such as GO, KEGG, EggNOG, NR, Swiss‐Prot, and Pfam. In total, there were 22,407 (96.14%) genes and 42,203 (97.35%) transcripts successfully annotated (Table 4).

**TABLE 4:** Functional annotation of transcriptome data in six public protein databases.

Type	Gene number (percent)	Transcript number (percent)
GO	15,807 (67.82%)	29,288 (67.56%)
KEGG	11,752 (50.42%)	22,913 (52.85%)
EggNOG	15,202 (65.23%)	29,353 (67.71%)
NR	22,403 (96.12%)	42,199 (97.34%)
Swiss‐Prot	14,751 (63.29%)	28,523 (65.79%)
Pfam	16,531 (70.93%)	31,275 (72.14%)
Total annotation	22,407 (96.14%)	42,203 (97.35%)
Total	23,307 (100.00%)	43,354 (100.00%)

**FIGURE 7:**
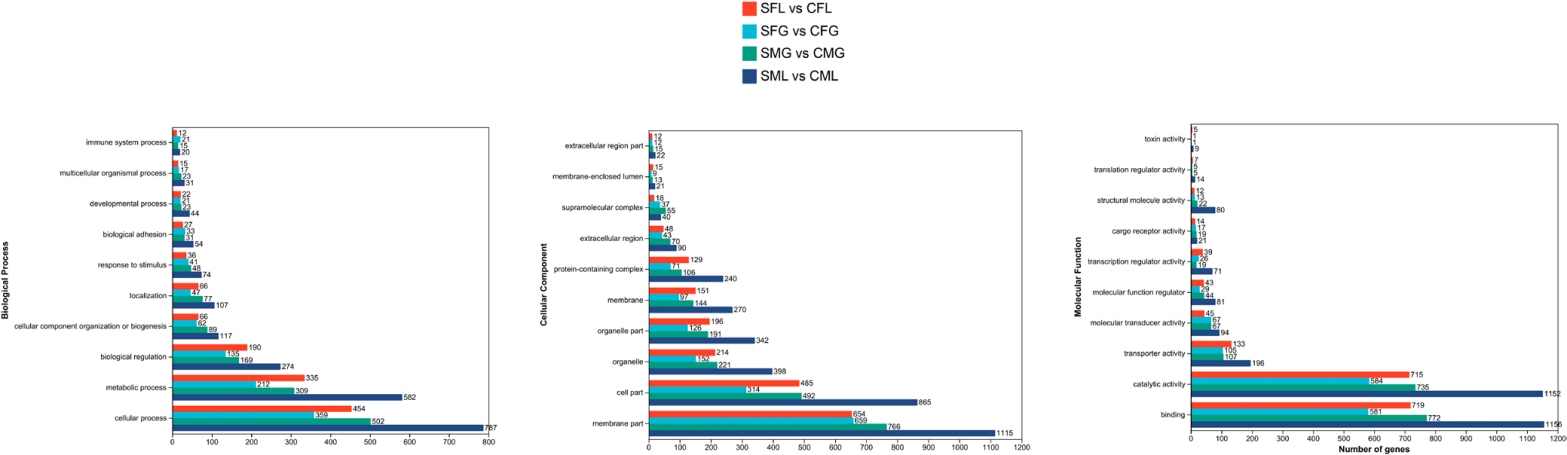
Histogram of GO classifications of *P. canaliculata* consensus sequences. Results are summarized for the three main GO categories: biological process, cellular component, and molecular function. The left axis indicates the number of genes in each category. SFL represents saline‐treated liver tissue of female snails; SML represents saline‐treated liver tissue of male snails; SFG represents saline‐treated gills tissue of female snails; SMG represents saline‐treated gills tissue of male snails; CFL represents without saline‐treated liver tissue of female snails; CML represents without saline‐treated liver tissue of male snails; CFG represents without saline‐treated gills tissue of female snails; CMG represents without saline‐treated gills tissue of male snails. Four replicates of each treatment were carried out in RNA‐seq analysis.

Transcriptome genes in the livers and gills of the male and female *P. canaliculata* were annotated and assigned into three categories: biological process (BP), cellular component (CC) and molecular function (MF). Among those assigned to the category of BP, in the following order as livers of the female snails (SFL vs. CFL), gills of the female snails (SFG vs. CFG), gills of the male snails (SMG vs. CMG) and livers of the male snails (SML vs. CML), cellular process (37.1%, 37.9%, 39.0%, 37.6%), metabolic process (27.4%, 22.4%, 24.0%, 27.8%), bioligical regulation (15.5%, 14.2%, 13.1%, 13.1%) were the highly represented. Among those assigned to the category of CC, membrane part (34.0%, 43.6%, 37.0%, 32.8%), cell part (25.2%, 20.6%, 23.7%, 25.4%) and oranelle (11.1%, 10.0%, 10.7%, 11.7%) were the highly represented. Among those assigned to the category of MF, binding (41.5%, 40.6%, 43.1%, 40.2%), catalytic activity (41.3%, 40.9%, 41.0%, 40.1%) and transporter activity (7.7%, 7.3%, 6.0%, 6.8%) were the highly represented (Figure 7).

**FIGURE 8:**
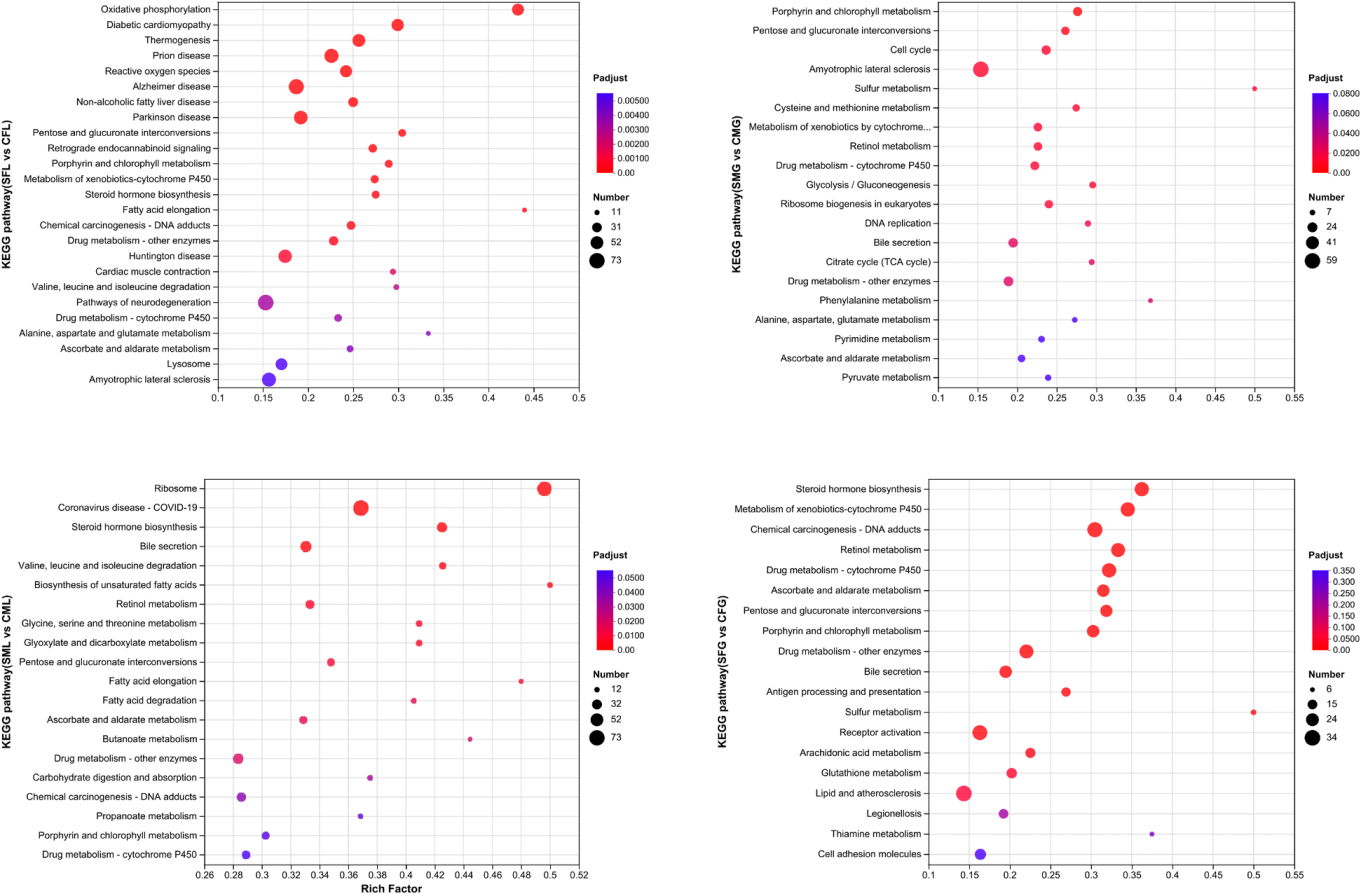
Scatter plot showing KEGG pathway enrichment among the DEGs. The vertical axis represents the pathway categories, and the horizontal axis shows the enrichment factor. The point size shows the number of DEGs among the pathway. The bigger the point size, the more genes in the pathway. SFL represents saline‐treated liver tissue of female snails; SML represents saline‐treated liver tissue of male snails; SFG represents saline‐treated gills tissue of female snails; SMG represents saline‐treated gills tissue of male snails; CFL represents without saline‐treated liver tissue of female snails; CML represents without saline‐treated liver tissue of male snails; CFG represents without saline‐treated gills tissue of female snails; CMG represents without salt‐treated gills tissue of male snails. Four replicates of each treatment were carried out in RNA‐seq analysis.

By employing KEGG pathway analysis, molecular interaction networks within cells can be identified, facilitating the elucidation of potential biological functions attributed to the analyzed genes (Figure 8).

In the livers of female snails (SFL vs. CFL), KEGG analysis revealed the significant enrichment of 25 pathways. Among these, the “Fatty acid elongation” pathway exhibited the highest rich factor, playing a crucial role in lipid metabolism and cellular energy balance. The “Pathways of neurodegeneration” showed the highest number of DEGs, suggesting potential neurophysiological stress responses under salinity conditions. In the gills of female snails (SFG vs. CFG), KEGG analysis identified 19 highly enriched pathways. The “Sulfur metabolism” pathway, which is critical for detoxification and oxidative stress regulation, demonstrated the highest rich factor. The “Chemical carcinogenesis—DNA adducts” pathway exhibited the largest number of DEGs, indicating possible DNA damage and repair mechanisms triggered by saline stress. In the gills of male snails (SMG vs. CMG), KEGG analysis revealed the significant enrichment of 16 pathways, the “Sulfur metabolism” pathway possessed the highest rich factor, reinforcing its role in stress adaptation. The “Amyotrophic lateral sclerosis” pathway had the most DEGs, suggesting that oxidative stress‐related neurodegenerative mechanisms may be involved in salinity adaptation. In the livers of male snails (SML vs. CML), KEGG analysis identified 19 highly enriched pathways, the “Biosynthesis of unsaturated fatty acids” pathway displayed the highest rich factor, which is essential for maintaining membrane fluidity and energy storage. The “Coronavirus disease—COVID‐19” pathway had the most DEGs, likely due to its broad association with immune response and cellular stress pathways in KEGG annotations.

**FIGURE 9:**
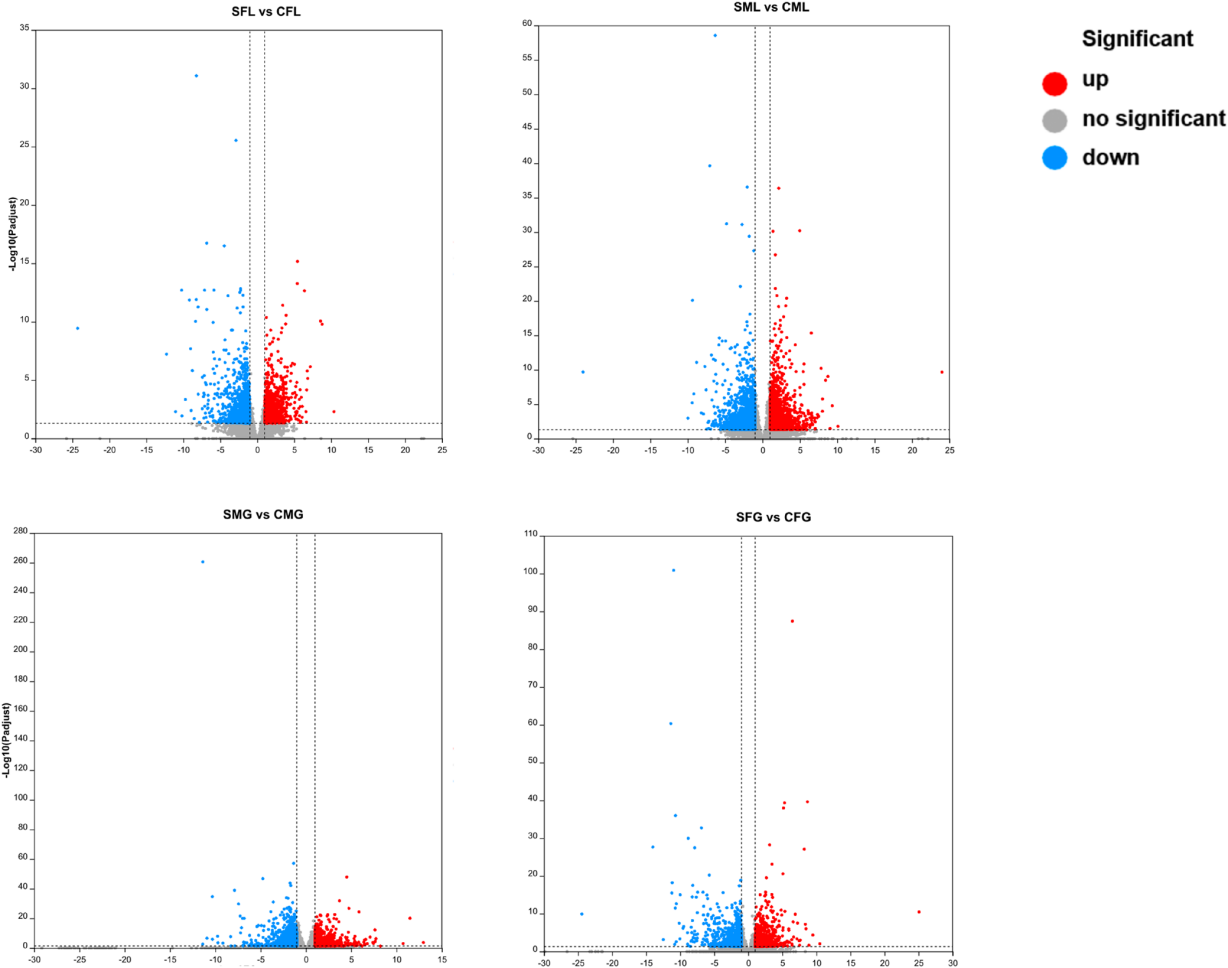
Volcano plot of DEGs in livers and gills of the male and female snails after salinity treatment. The data for all genes were plotted as log2 fold change versus the –log10 of the adjusted *p*‐value. Significant differentially expressed genes comparing different treatments were highlighted in red dot (upregulation) and blue (downregulation), while genes with no significant differences were drawn in gray. SFL represents saline‐treated liver tissue of female snails; SML represents saline‐treated liver tissue of male snails; SFG represents saline‐treated gills tissue of female snails; SMG represents saline‐treated gills tissue of male snails; CFL represents without saline‐treated liver tissue of female snails; CML represents without saline‐treated liver tissue of male snails; CFG represents without saline‐treated gills tissue of female snails; CMG represents without saline‐treated gills tissue of male snails. Four replicates of each treatment were carried out in RNA‐seq analysis.

Pairwise comparisons were conducted to identify DEGs between the 2 ppt salinity treatment group and the control group. In the gills and livers of female and male snails, 22,215, 22,284, 22,314, and 21,984 genes, respectively, met the criteria of having a fold change greater than 2 and an adjusted *p* < 0.05. In the comparison of the same organ, both the upregulation and the downregulation of DEGs were greater in males than in females after salt stress. In a comparison of different organs of the same sex, the proportion of changes in DEGs following liver response to stress was greater than that of gills, with the greatest upregulation and downregulation of DEGs in male liver (Figure 9).

### 3.10 Impact of Saline Stress on Hatchability and Inhibition Rate of Eggs

**FIGURE 10:**
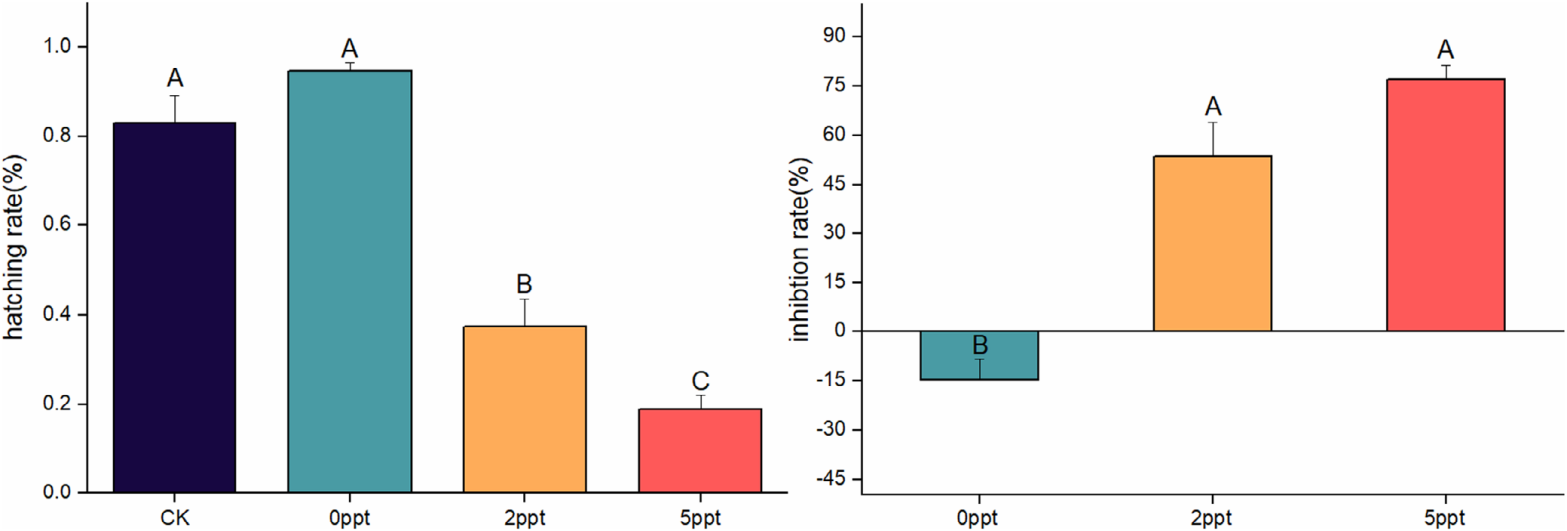
Hatchability and inhibition rate of *P. canaliculata* eggs under saline stress. CK represents the condition that involved no liquid application, simulating the natural hatching conditions of eggs in the environment. The error bars represent the standard error. Different capital letters indicate significant differences (*p* < 0.05) between treatments.

In the collected experimental samples, each egg mass exhibited varying degrees of hatching. The hatching rate decreased with increasing salinity of the treatments, while the hatching inhibition rate increased with the higher salinity levels. Among all the egg mass samples, the average hatching rate of all eggs at the end of the experiment was 59.0%. As depicted in Figure 10, the average hatching rate was 83.0% in the control group (CK), with the highest average observed in the 0 ppt group at 94.7%. The hatching rate in the 0 ppt group was significantly higher than that in the other two saline treatment groups, with a rate of 37.3% at 2 ppt treatment and the lowest average rate of 19.0% at 5 ppt treatment. Using the CK group as a reference, the experiment revealed that 0 ppt had a promotive effect on the hatching of eggs, with a promotion rate of 14.7%. Conversely, at a salinity of 2 ppt treatment, the hatching of eggs was significantly inhibited, with an inhibition rate of 53.5%. At the highest concentration treatment, the inhibition rate reached 77.0%, indicating that salinity has a certain inhibitory effect on the hatching of eggs (Figure 10).

### 3.11 Impact of Saline Stress on Physiological and Biochemical Substances of Eggs

**FIGURE 11:**
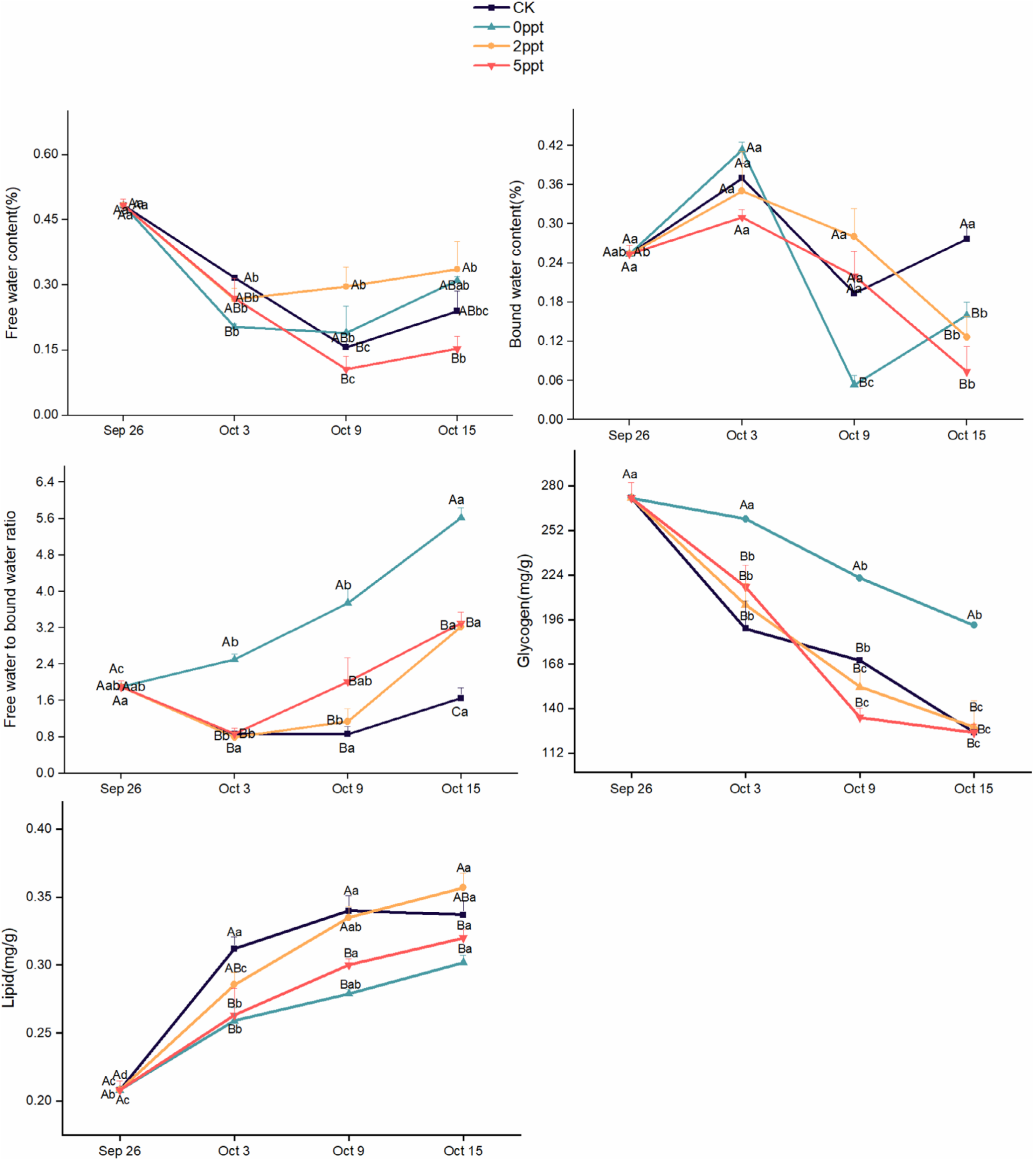
Physiological and biochemical substance content of *P. canaliculata* eggs under long‐term saline stress treatments. CK represents the condition that involved no liquid application, simulating the natural hatching conditions of *P. canaliculata* eggs in the environment. The error bars represent the standard error. Capital letters indicate differences between the treatments; lowercase letters indicate differences in treatment duration; different letters represent significant intergroup differences (*p* < 0.05).

The free and bound water contents of eggs after the saline stress treatment were determined. The results revealed that by the 7th day of the experiment, the free water content in all four treatments significantly decreased. In the CK group, the absence of stress resulted in a reduction in free water levels due to the involvement of free water in various physiological activities during egg hatching. The decline of the 5 ppt group was the most dramatic. The bound water content in the CK group remained unchanged throughout the experiment. According to the trends in free and bound water content across different treatment groups (as shown in the Figure 11), the ratio of free water to bound water in the 0 ppt group increased gradually over the course of the experiment, while the ratio in the 2 and 5 ppt groups rose slowly from 7th day to the end of the experiment. The ratio in the CK group remained constant throughout the study. Among the four treatment groups, glycogen content in the 0 ppt group significantly decreased by 13th day and was higher than in the other three groups. Glycogen levels in the other groups also significantly decreased by 7th day, with no significant differences among them until the end of the experiment. Fat content increased progressively in all treatment groups, with the 2 ppt group showing the most pronounced increase in fat content over the course of the experiment (Figure 11).

### 3.12 Impact of Saline Stress on Antioxidant Enzymes of Eggs

**FIGURE 12:**
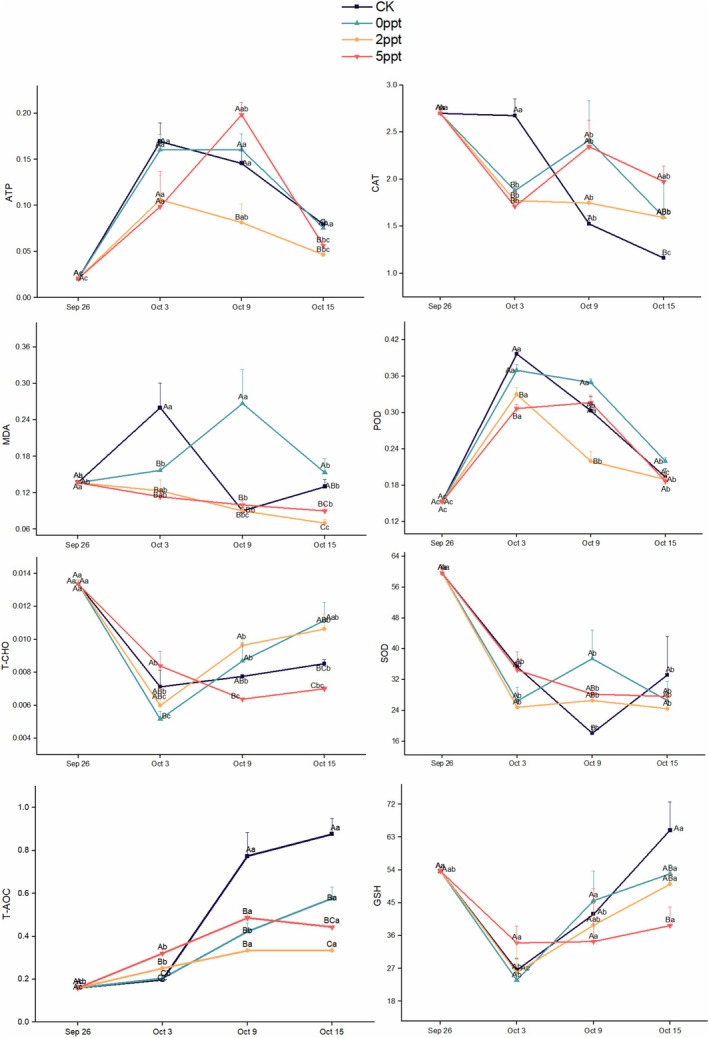
Antioxidative system indices of *P. canaliculata* eggs under long‐term saline stress. CK represents the condition that involved no liquid application, simulating the natural hatching conditions of eggs in the environment. The error bars represent the standard error. Capital letters indicate differences between treatments; lowercase letters indicate differences in treatment duration; different letters represent significant intergroup differences (*p* < 0.05). All biochemical parameters were normalized to protein content. ATP is expressed as nmol/mg protein. CAT, POD, SOD, and T‐AOC are expressed as U/mg protein. MDA is expressed as nmol/mg protein. GSH is expressed as μmol/mg protein. T‐CHO is expressed as μg/mg protein.

Various oxidative stress‐related indicators of eggs at different stages were determined. Except for the CK group, ATPase activity in all treatment groups initially increased and then decreased throughout the experiment. On the final day, ATPase activity was significantly lower in the 2 and 5 ppt treatment groups compared with the CK and 0 ppt groups. CAT activity in the CK group decreased from 13th day onward and continued to decline until the end of the experiment, showing a significant difference from the 5 ppt group, while other groups exhibited minimal enzyme activity changes. In both the CK and 0 ppt groups, which were not subjected to saline stress, MDA content showed an overall trend of initial increase followed by a decrease, whereas saline treatment led to a reduction in MDA content in the egg masses. POD activity increased significantly in all treatment groups from the start to 7th day, with CK and 0 ppt groups showing notably higher POD activity compared with the 2 and 5 ppt groups. From 7th day to the end of the experiment, POD activity gradually returned to baseline levels. T‐CHO content was significantly lower than the initial value on 7th day for all treatment groups, with CK, 0, and 2 ppt groups showing a gradual increase in T‐CHO content, while the 5 ppt group had the lowest T‐CHO content on 13th day. SOD activity significantly decreased early in the experiment and then stabilized, with all groups showing stable SOD activity after 7th day. T‐AOC activity increased throughout the experiment in all treatment groups, with the CK group showing significantly higher levels than the other groups from 13th day onward. GSH activity in the CK, 0 ppt, and 2 ppt groups exhibited an initial increase followed by a decrease during the experiment period (Figure 12).

## 4 Discussion

**FIGURE 13:**
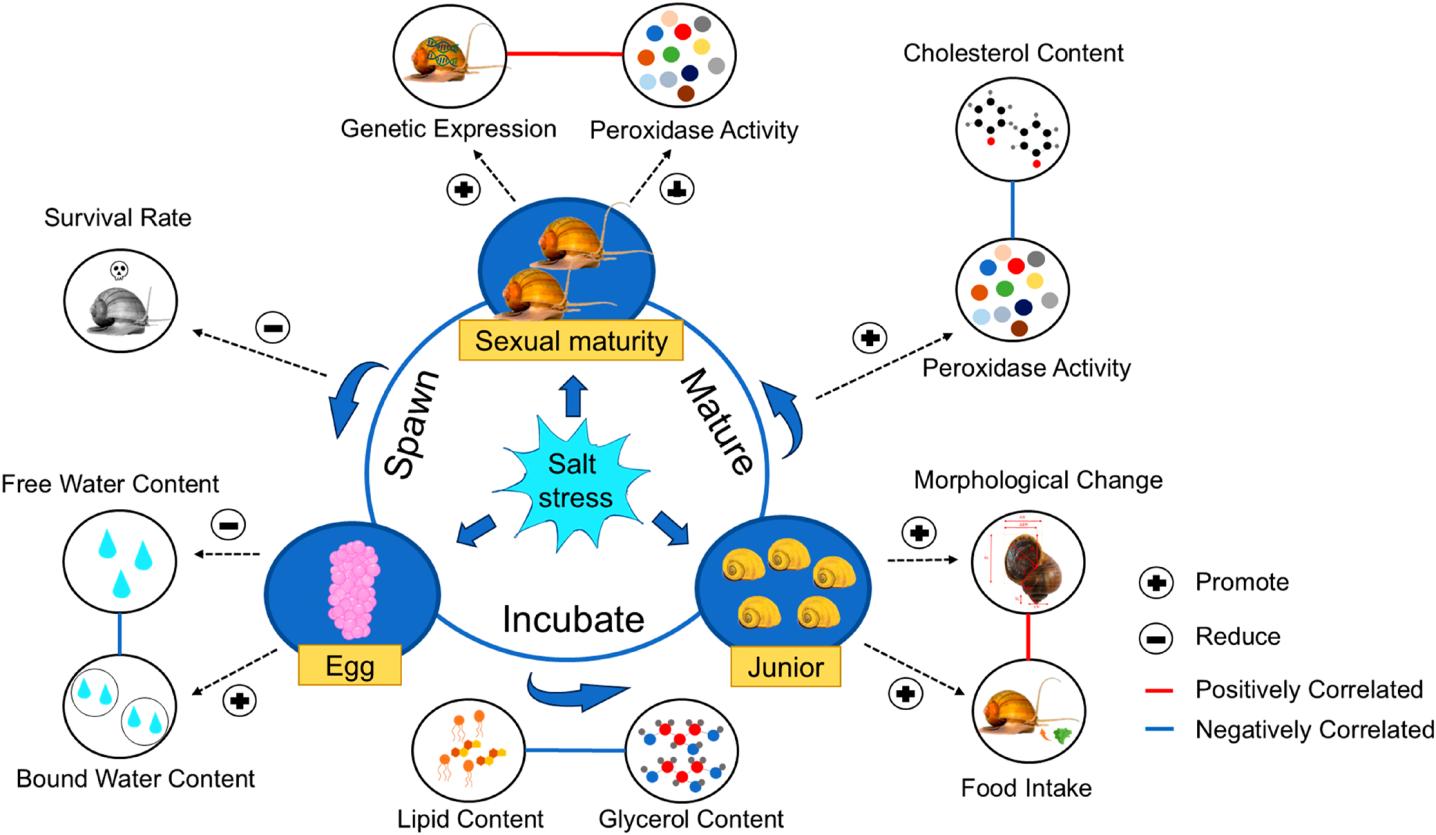
Schematic diagrams showing changes in physiology of *P. canaliculata* under saline stress treatments, based on the results obtained.

The exposure to low‐concentration saline treatment significantly reduced the survival rate of *P. canaliculata* in the early stages (Figure 13). As the salinity increased and the duration of exposure extended, the survival rate gradually declined (Figure 2). This initial lack of defense mechanisms against external stress explains the observed decrease in survival. However, in the later stages, the survival rate approached that of the control group, indicating the snails had an ability to acclimate to the changing salinity. This adapation may involve the regulation of intracellular ion concentrations and the synthesis and secretion of osmoregulatory substances. Accumulation of these substances helps maintain osmotic balance and enables the snails to cope with saline stress. This gradual convergence of survival rates with the control group suggests an acclimatory response to the stressors. In addition, saline stress may induce changes in the energy metabolism of *P. canaliculata* to meet the demands of the changing environment (Liu, Liu, et al. 2022; Purwaningsih et al. 2015). Studies have shown that mollusks can maintain their internal energy levels in low‐saline environments through increased feeding and reduced metabolic rates (Liu, Liu, Zhao, Li, et al. 2022). This metabolic acclimation likely contributes to the gradual reduction of the mortality rate in *P. canaliculata* under long‐term low‐concentration saline stress (Bassler‐Veit et al. 2013; Carral et al. 2023). Consequently, in this experiment, the snails subjected to saline stress exhibited significantly higher food intake and body weights compared with the control group.

After saline stress treatment, the shell strength and thickness of the snails increased, and there was a significant widening of the overall shell morphology (Figures 3 and 4). The reason for these changes may involve an increase in calcium deposition since the snails face higher concentrations of calcium ions in saline environments (Vokhshoori et al. 2023; Fernández et al. 2023). To adapt to this environment, snails may increase the rate of calcium deposition, making the shell tissue harder and stronger (Casado‐Coy et al. 2022). This can also trigger the synthesis of shell proteins, promoting shell formation (Li et al. 2023; Hu et al. 2021), and allowing for greater energy reserve storage within the shell to withstand the stress (Garcia et al. 2021). It is worth mentioning that saline environments may impose higher demands on the snail shell, such as increased strength and protective functions, to cope with more adverse environmental conditions (Thakur et al. 2021). Thickening and strengthening of the shell provide better protection and support, helping the snail with stand external pressures and predation threats (Alati et al. 2020; Sakalauskaite et al. 2020).

In the present study, transcriptomic analysis revealed that the highest content in the “Biological Process” category was attributed to the regulation and response of various cellular processes at the cellular level, which were induced by the saline treatment. When exposed to a saline environment, aquatic organisms accumulate specific organic solutes such as glycerol and proline to increase the intracellular osmotic pressure, thereby attracting water molecules into the cells to prevent cellular dehydration (Ma et al. 2019). They also regulate the expression and activity of ion channels and transport proteins for ions like Na^+^, K^+^, and Ca^2+^ to control intracellular ion concentrations (Iqbal et al. 2023). Additionally, the activity of antioxidant enzymes undergoes corresponding changes to eliminate reactive oxygen species and neutralize free radicals, while some proteins undergo abnormal folding and degradation (Serba et al. 2016). In this study, the levels of CAT increased under saline stress, which aided in the breakdown of hydrogen peroxide. Concurrently, the upregulation of SOD activity played a crucial role in scavenging superoxide anions (O^2−^), while the overall antioxidant capacity was enhanced. The regulation of ACHE activity helped maintain neural transmission and adapt to environmental changes. The observed decrease in MDA levels was attributed to the strengthened antioxidant system, which effectively reduced lipid peroxidation. Additionally, the reduction in NOS activity likely minimized the production of reactive nitrogen species, thereby mitigating cellular damage (Figure 5). Under conditions of long‐term saline stress, the liver and gill tissues of *P. canaliculata* need to acclimate to the cellular environmental changes induced by saline stress by regulating various cellular processes (Gao et al. 2022).

Studies have shown that mollusks can increase the activity of antioxidant enzymes to reduce oxidative stress on cells (Cao et al. 2020; Zhang et al. 2017). The changes in the lipid composition of the cell membrane and the activation of cellular regulatory mechanisms are caused by saline stress (Xiong et al. 2018). This leads to an increase in the expression of genes related to lipid synthesis to enhance membrane lipid production, the modulation of genes related to lipid metabolism to adjust the rate of membrane lipid synthesis and metabolism, and the regulation of protein synthesis and modification to acclimate to changes in membrane composition and adjust the structure and function of the cell membrane (Liu et al. 2020). These adjustments aim to maintain the stability of the intracellular and extracellular environments, regulate the membrane potential, and ensure proper cellular excitability (Liu, Li, et al. 2022). In the GO analysis of this experiment, it was found that the cellular membrane components of liver cells and protein expression levels were significantly elevated after saline stress (Figure 7).

Saline treatment induced various cellular functional and metabolic changes. These changes primarily involve the regulation of metabolic enzymes to acclimate to alterations in energy metabolism (Wang et al. 2022; Ma et al. 2018). The expression and functionality of binding proteins, such as receptors and signal transduction molecules, are modulated to accommodate changes in signal transduction (Zhou et al. 2020). Under saline stress, freshwater snails may initiate a series of cellular protective mechanisms to mitigate damage to cell structure and function (Si et al. 2018; Cui et al. 2019). The results of the volcano plot analysis revealed that the majority of significantly differentially expressed genes, both in the liver and gills, were associated with metabolism. Among the highly expressed metabolism‐related pathways in these tissues were “Metabolism of xenobiotics‐cytochrome P450”, “Drug metabolism‐cytochrome P450”, and “Alanine, aspartate and glutamate metabolism” (Figure 8). Saline stress may affect the sulfur metabolism pathway in the gills of snails, as the gills are involved in nitrogen metabolism processes, regulating the expression and activity of sulfite reductases, as well as adjusting the synthesis and degradation rates of sulfates (Sharma et al. 2019; Planells et al. 2019; Al‐Tobasei et al. 2017). At the same time, oxidative stress, apoptosis, and inflammation occurred, leading to abnormal gene expression related to ALS in the gill tissue (Li et al. 2018). When aquatic animals are exposed to carcinogenic substances in the environment, these substances can enter their bodies through gill tissues. The gills participate in the metabolism and detoxification processes of carcinogenic substances through metabolic reactions. DNA can be damaged by environmental factors and chemical substances, including DNA damage caused by carcinogenic agents. Gill cells possess various DNA repair mechanisms to correct and repair DNA damage, including base repair, nucleotide repair, and DNA strand break repair. These repair processes help protect the integrity of DNA and prevent cell mutations and carcinogenesis caused by DNA damage induced by carcinogens (Quigley et al. 2016; Buselic et al. 2018; Gao et al. 2018). Following saline stress, the liver exhibited more upregulated and downregulated DEGs than gills, indicating differences in structure, function, and metabolism (Figure 9). This may be attributed to the liver's important role as a metabolic organ responsible for substance metabolism and detoxification in the snail. It is involved in multiple metabolic pathways. Therefore, when facing stressful conditions, the liver may be more sensitive to metabolic regulation in order to cope with changes in energy metabolism and metabolic balance compared with the gills (Swinehart et al. 1998; Cheney et al. 2008). Due to its rich metabolic activity and involvement in oxygen metabolism processes, the liver may be more susceptible to oxidative stress. The liver is also an important site for immune response, with abundant distribution of immune cells and immune factors (Pan and Han 2023; Wang et al. 2023). Under stress conditions, the snail liver may initiate an immune response to counter potential pathogen invasion and infection (Chen et al. 2022; Martemyanov et al. 2021). This may lead to the regulation of immune‐related genes in the liver, making it more sensitive to stress (Jeyavani et al. 2022; Anagha et al. 2022; Chakraborty and Joy 2020). In the liver of male snails, genes related to fatty acid synthesis were significantly differentially expressed under saline treatment conditions. The snails may require adjustments in the fatty acid synthesis pathway to acclimate to changes in the stability and functional demands (Pan et al. 2019). The process of fatty acid elongation involves the participation of multiple enzymes and substrates in the synthesis of long‐chain fatty acids within cells (Wang et al. 2018). This regulatory response may be aimed at acclimation to the energy demands and metabolic adjustments under saline stress conditions (Zhang et al. 2018).

The volcano plot analysis revealed that the number of upregulated and downregulated DEGs in male snails after saline stress was higher than in female snails, indicating differences in gene expression patterns between male and female individuals of the snail species (Figure 9). The regulation of genes and hormones may lead to different transcriptional responses to saline stress in male and female individuals, as well as different physiological acclimatory capacities to cope with saline stress (Xu et al. 2019). These DEGs may be involved in pathways related to cell membrane stability, osmotic regulation, ion balance, and cellular stress (Slattery et al. 2018). Male and female snails may have sex‐specific gene regulatory networks, which may include sex‐determining genes, sex hormone receptors, and sex‐specific transcription factors (Yang et al. 2023; Liu, Li, et al. 2022; Li and Zou 2019). Long‐term saline treatment may impact the snail's nervous system, leading to an increased number of DEGs in this pathway (Serba et al. 2016). Saline stress could trigger cellular disturbances such as oxidative stress and inflammation, which may have adverse effects on the nervous system. Additionally, saline treatment may affect the synthesis of neurotransmitters, neuronal function, and interactions between nerve cells, resulting in an increased number of DEGs in the “Pathways of neurodegeneration” (Sharma et al. 2019).

In a saline environment, *P. canaliculata* eggs undergo metabolic adjustments to acclimate to the new conditions. Salinity has a negative impact on egg hatching, as increased salinity raises osmotic pressure, leading to water loss. As salinity levels rise, the increasing osmotic pressure increases the dehydration of the eggs. Excessive dehydration and deformation of the eggs disrupt their normal hatching process, resulting in lower hatching rates (Wang et al. 2012). This experiment also confirmed that higher saline concentrations in the water environment correlated with lower hatching rates for *P. canaliculata* eggs. The 0 ppt condition exhibited the highest hatching rate and the highest free water to bound water ratio; it also had the highest glycogen reserves. Compared with the control, the 0 ppt group further promoted the development of egg masses, suggesting that it helps maintain the normal shape of the eggs, prevents dehydration, and thus facilitates the hatching process (Figure 10).

The saline environment negatively affects the stability of egg cell membranes, which are critical for maintaining cellular structure and function. High salinity can cause membrane rupture or surface macromolecule denaturation, disrupting embryonic development. Additionally, elevated saline concentrations increase metabolic stress on the eggs. In response to hyperosmotic conditions, embryos may require more energy to sustain normal physiological processes, potentially altering their growth and developmental cycles (Dreon et al. 2007; Salleh and Arbain 2015; Liu et al. 2023). Our experiment revealed that high‐concentration saline stress reduced the total cholesterol content in *P. canaliculata* eggs, with cholesterol likely being utilized to address membrane damage caused by stress. Cholesterol also plays a role in modulating membrane fluidity, enabling cells to better acclimate to high‐salinity environments. Furthermore, compared with the control group, the variations in MDA and POD levels during the entire hatching period under high saline conditions indicated that high salinity altered the developmental trajectory of the eggs (Figure 11).

## 5 Conclusion

This study conducted a comprehensive analysis of the survival rates, physiological and biochemical characteristics, and transcriptomes of *P. canaliculata* across different growth stages to saline stress, as well as the related response mechanisms to saline stress. The results indicated that early survival rates significantly declined under saline stress, but the snails were able to maintain energy balance by increasing food intake and reducing metabolic rates, eventually reaching a survival rate close to that of the control group. *P. canaliculata* exhibited enhanced shell strength, thickness, and significant shell widening through increased calcium content and shell protein synthesis under saline stress, which improved their self‐protective functions. Additionally, changes in antioxidant enzyme activity displayed sexual dimorphism. Saline stress caused the regulation of cellular processes, where the accumulation of organic solutes and the modulation of ion channels were key mechanisms for acclimating to osmotic changes and preventing cellular damage. The liver and gills showed abundant expression of metabolism‐related pathways, with the liver being more sensitive to metabolic and immune responses than the gills. Saline stress also had negative effects on the eggs, with hatching rates decreasing as salinity levels increased. Moreover, saline stress negatively impacted membrane stability and metabolism, reducing total cholesterol content in the eggs and altering their developmental process. These findings not only provide a theoretical basis for the invasion mechanisms of *P. canaliculata* but also reveal the physiological, biochemical, and molecular acclimation strategies which the species employs in response to saline stress.

## Author Contributions

**Yingtong Chen:** conceptualization (equal), data curation (lead), formal analysis (lead), investigation (lead), methodology (lead), validation (lead), visualization (lead), writing – original draft (lead). **Fucheng Yao:** inverstigation (equal), methodolgy (equal). **Zhaoji Shi:** methodolgy (equal), software (equal), visualization (equal). **Chunxia Zhang:** investigation (equal). **Jimin Liu:** investigation (equal). **Jiaen Zhang:** conceptualization (lead), writing‐review and editing (lead), project adminstration (lead), resources (lead), funding acquisition (lead), supervision (lead). **Zhong Qin:** methodolgy (equal), validation (equal).

## Conflicts of Interest

The authors declare no conflicts of interest.

## Data Availability

The raw data are available in SRA (https://www.ncbi.nlm.nih.gov/sra) under BioProject numbers PRJNA1178854. The transcriptomic data were analyzed using a reference genome obtained from the public NCBI database.

## References

[ece371581-bib-0001] Alati, V. M. , J. Olunga , M. Olendo , et al. 2020. “Mollusc Shell Fisheries in Coastal Kenya: Local Ecological Knowledge Reveals Overfishing.” Ocean and Coastal Management 195: 105285.

[ece371581-bib-0002] Al‐Tobasei, R. , A. Ali , T. D. Leeds , et al. 2017. “Identification of SNPs Associated With Muscle Yield and Quality Traits Using Allelic‐Imbalance Analyses of Pooled RNA‐Seq Samples in Rainbow Trout.” BMC Genomics 18, no. 1: 582. 28784089 10.1186/s12864-017-3992-z PMC5547479

[ece371581-bib-0003] Anagha, B. , P. S. Athira , P. Anisha , P. E. Charles , A. Anandkumar , and R. Rajaram . 2022. “Biomonitoring of Heavy Metals Accumulation in Molluscs and Echinoderms Collected From Southern Coastal India.” Marine Pollution Bulletin 184: 114169. 36201985 10.1016/j.marpolbul.2022.114169

[ece371581-bib-0004] Andreeva, S. I. , N. I. Andreev , and R. A. Mikhaylov . 2020. “Records of the Mollusk Genus Caspiohydrobia Starobogatov 1970 (Gastropoda, Hydrobiidae) in Salt Rivers of the Caspian Lowland.” Zoologicheskiĭ Zhurnal 99, no. 3: 253–260.

[ece371581-bib-0005] Bassler‐Veit, B. , I. F. Barut , E. Meric , et al. 2013. “Distribution of Microflora, Meiofauna, and Macrofauna Assemblages in the Hypersaline Environment of Northeastern Aegean Sea Coasts.” Journal of Coastal Research 29, no. 4: 883–898.

[ece371581-bib-0006] Batista, F. M. , R. Hatfield , A. Powell , C. Baker‐Austin , J. Lowther , and A. D. Turner . 2023. “Methodological Advances in the Detection of Biotoxins and Pathogens Affecting Production and Consumption of Bivalve Molluscs in a Changing Environment.” Current Opinion in Biotechnology 80: 102896. 36773575 10.1016/j.copbio.2023.102896

[ece371581-bib-0007] Buselic, I. , Ž. Trumbić , J. Hrabar , A. Vrbatović , I. Bočina , and I. Mladineo . 2018. “Molecular and Cellular Response to Experimental Anisakis Pegreffii (Nematoda, Anisakidae) Third‐Stage Larval Infection in Rats.” Frontiers in Immunology 9: 2055. 30245697 10.3389/fimmu.2018.02055 PMC6137129

[ece371581-bib-0008] Cao, D. , J. Li , B. Huang , et al. 2020. “RNA‐Seq Analysis Reveals Divergent Adaptive Response to Hyper‐ and Hypo‐Salinity in Cobia, *Rachycentron canadum* .” Fish Physiology and Biochemistry 46, no. 5: 1713–1727. 32514851 10.1007/s10695-020-00823-7

[ece371581-bib-0009] Carral, L. , M. I. Lamas‐Galdo , J. L. M. Buenhombre , J. J. C. Barros , S. Naya , and J. Tarrio‐Saavedra . 2023. “Application of Residuals From Purification of Bivalve Molluscs in Galician to Facilitate Marine Ecosystem Resiliency Through Artificial Reefs With Shells—One Generation.” Science of the Total Environment 856: 159095. 36181815 10.1016/j.scitotenv.2022.159095

[ece371581-bib-0010] Casado‐Coy, N. , P. Sánchez‐Jerez , J. S. Troncoso , and C. Sanz‐Lazaro . 2022. “Mollusc‐Shell Debris Derived From Aquaculture Can Promote Macrofaunal Communities With a High Bioturbation Capacity.” Aquaculture 548: 737642.

[ece371581-bib-0011] Catalan, M. , M. S. Dreon , H. Heras , R. J. Pollero , and S. N. Fernández . 2006. “Pallial Oviduct of *Pomacea canaliculata* (Gastropoda): Ultrastructural Studies of the Parenchymal Cellular Types Involved in the Metabolism of Perivitellins.” Cell and Tissue Research 324, no. 3: 523–533. 16453107 10.1007/s00441-005-0132-x

[ece371581-bib-0012] Chakraborty, K. , and M. Joy . 2020. “High‐Value Compounds From the Molluscs of Marine and Estuarine Ecosystems as Prospective Functional Food Ingredients: An Overview.” Food Research International 137: 109637. 33233216 10.1016/j.foodres.2020.109637 PMC7457972

[ece371581-bib-0013] Chen, L. , X. Cai , M. Cao , et al. 2022. “Long‐Term Investigation of Heavy Metal Variations in Mollusks Along the Chinese Bohai Sea.” Ecotoxicology and Environmental Safety 236: 113443. 35364504 10.1016/j.ecoenv.2022.113443

[ece371581-bib-0014] Cheney, M. A. , D. Keil , and S. Qian . 2008. “Uptake and Effect of Mercury on Amino Acid Losses From the Gills of the Bivalve Mollusks Mytilus Californianus and *Anodonta californiensis* .” Journal of Colloid and Interface Science 320, no. 2: 369–375. 18272165 10.1016/j.jcis.2007.12.045

[ece371581-bib-0015] Corbin, T. , A. Accorsi , B. Pardo , et al. 2022. “CRISPR/Cas9 Technology Opens Door to Genetic Manipulations in the Freshwater Apple Snail, *Pomacea canaliculata* .” Transgenic Research 31, no. Suppl 1: 28.

[ece371581-bib-0016] Cui, Q. , L. Qiu , X. Yang , et al. 2019. “Transcriptome Profiling of the Low‐Salinity Stress Responses in the Gills of the Juvenile *Pseudopleuronectes yokohamae* .” Comparative Biochemistry and Physiology. Part D, Genomics & Proteomics 32: 100612. 10.1016/j.cbd.2019.100612 31387066

[ece371581-bib-0017] Dreon, M. S. , M. Ceolin , and H. Heras . 2007. “Astaxanthin Binding and Structural Stability of the Apple Snail Carotenoprotein Ovorubin.” Archives of Biochemistry and Biophysics 460, no. 1: 107–112. 17324373 10.1016/j.abb.2006.12.033

[ece371581-bib-0018] Fernández, L. , F. Ruiz , G. Gómez , et al. 2023. “Mollusc Collection and Holocene Palaeogeographical Evolution in a Southwestern Iberian Estuary: Statistical Analysis of the Early Holocene Cañada Honda Shell Midden (SW Spain).” Quaternary International 658: 24–35.

[ece371581-bib-0019] Gaiser, E. E. , A. Zafiris , P. L. Ruiz , F. A. C. Tobias , and M. S. Ross . 2006. “Tracking Rates of Ecotone Migration due to Salt‐Water Encroachment Using Fossil Mollusks in Coastal South Florida.” Hydrobiologia 569, no. 1: 237–257.

[ece371581-bib-0020] Gao, M. , K. Wang , M. Yang , et al. 2018. “Transcriptome Analysis of Bronchoalveolar Lavage Fluid From Children With *Mycoplasma pneumoniae* Pneumonia Reveals Natural Killer and T Cell‐Proliferation Responses.” Frontiers in Immunology 9: 1403. 29967623 10.3389/fimmu.2018.01403 PMC6015898

[ece371581-bib-0021] Gao, Y. , J. N. Li , J. J. Pu , K. X. Tao , X. X. Zhao , and Q. Q. Yang . 2022. “Genome‐Wide Identification and Characterization of the HSP Gene Superfamily in Apple Snails (Gastropoda: Ampullariidae) and Expression Analysis Under Temperature Stress.” International Journal of Biological Macromolecules 222: 2545–2555. 36228823 10.1016/j.ijbiomac.2022.10.038

[ece371581-bib-0022] Garcia, M. F. L. , A. J. M. Araújo , R. A. Raimundo , R. M. Nascimento , J. P. F. Grilo , and D. A. Macedo . 2021. “Electrical Properties of ca‐Doped Ceria Electrolytes Prepared by Proteic Sol‐Gel Route and by Solid‐State Reaction Using Mollusk Shells.” International Journal of Hydrogen Energy 46, no. 33: 17374–17387.

[ece371581-bib-0023] Hu, H.‐P. , J.‐L. Feng , J.‐H. Liu , et al. 2021. “Palaeo‐Hydrochemistry Reconstructed From Fossil Mollusc Shells From Dammed Palaeo‐Lake Sediments in the Yarlung Tsangpo Valley, Tibet.” Applied Geochemistry 132: 105069.

[ece371581-bib-0024] Iqbal, A. , D. Qiang , W. Xiangru , et al. 2023. “Integrative Physiological, Transcriptome and Metabolome Analysis Reveals the Involvement of Carbon and Flavonoid Biosynthesis in Low Phosphorus Tolerance in Cotton.” Plant Physiology and Biochemistry 196: 302–317. 36738510 10.1016/j.plaphy.2023.01.042

[ece371581-bib-0025] Jeyavani, J. , A. Sibiya , N. Gopi , S. Mahboob , M. N. Riaz , and B. Vaseeharan . 2022. “Dietary Consumption of Polypropylene Microplastics Alter the Biochemical Parameters and Histological Response in Freshwater Benthic Mollusc *Pomacea paludosa* .” Environmental Research 212: 113370. 35504343 10.1016/j.envres.2022.113370

[ece371581-bib-0026] Koudenoukpo, Z. C. , O. H. Odountan , P. A. Agboho , et al. 2021. “Using Self–Organizing Maps and Machine Learning Models to Assess Mollusc Community Structure in Relation to Physicochemical Variables in a West Africa River–Estuary System.” Ecological Indicators 126: 107706.

[ece371581-bib-0027] Li, S. , Y. Xuan , B. Gao , et al. 2018. “Identification of an Eight‐Gene Prognostic Signature for Lung Adenocarcinoma.” Cancer Management and Research 10: 3383–3392. 30237740 10.2147/CMAR.S173941 PMC6138967

[ece371581-bib-0028] Li, S. , and Z. Zou . 2019. “Toxicity of Chimonanthus Nitens Flower Extracts to the Golden Apple Snail, *Pomacea canaliculata* .” Pesticide Biochemistry and Physiology 160: 136–145. 31519248 10.1016/j.pestbp.2019.07.015

[ece371581-bib-0029] Li, Y. , S. Liang , H. Ji , and X. Li . 2023. “Effect of Organic Matrix Viscoelasticity on Indentation Recovery Behavior in a Crossed‐Lamellar Structure of Mollusk Shell.” Materials Letters 330: 133391.

[ece371581-bib-0030] Liu, J. , J. Li , Z. Wang , and H. Yang . 2022. “Transcriptome Sequencing and Bioinformatics Analysis of Ovarian Tissues From *Pomacea canaliculata* in Guangdong and Hunan.” Mediators of Inflammation 2022: 3917036. 35431656 10.1155/2022/3917036 PMC9007660

[ece371581-bib-0031] Liu, J. , Z. Sun , Z. Wang , and Y. Peng . 2020. “A Comparative Transcriptomics Approach to Analyzing the Differences in Cold Resistance in *Pomacea canaliculata* Between Guangdong and Hunan.” Journal of Immunology Research 2020: 8025140. 32832573 10.1155/2020/8025140 PMC7422425

[ece371581-bib-0032] Liu, J. L. , J. Liu , B. Zhao , et al. 2022. “Palatability of Mangrove Leaves to Invasive Apple Snails: The Relation Between Feeding Electivity and Multiple Plant Characteristics.” Aquatic Invasions 17, no. 2: 277–299.

[ece371581-bib-0033] Liu, P. Y. , P. Liu , B. Zhao , et al. 2022. “Responses of Survival, Growth, and Feeding of the Invasive Golden Apple Snail (*Pomacea canaliculata*) to Salinity Stress.” Freshwater Science 41: 376–385.

[ece371581-bib-0034] Liu, Y. , X. Yang , Y. Chi , and Y. Chi . 2023. “Effects of CaCl2 on the Rheology, Microstructure and Protein Structures of Rapidly Salted Separated Egg Yolk.” Food Research International 172: 113096. 37689867 10.1016/j.foodres.2023.113096

[ece371581-bib-0035] Ma, B. , Z. Ran , X. Xu , et al. 2019. “Comparative Transcriptome Analyses Provide Insights Into the Adaptation Mechanisms to Acute Salt Stresses in Juvenile Sinonovacula Constricta.” Genes & Genomics 41, no. 5: 599–612. 30840180 10.1007/s13258-019-00805-x

[ece371581-bib-0036] Ma, Q. , C. Chen , Z. Zeng , et al. 2018. “Transcriptomic Analysis Between Self‐ and Cross‐Pollinated Pistils of Tea Plants ( *Camellia sinensis* ).” BMC Genomics 19, no. 1: 289. 29695246 10.1186/s12864-018-4674-1 PMC5918555

[ece371581-bib-0037] Ma, Y. , H. Chen , C. Yang , et al. 2023. “Validation of Target Protein PcnWAS in Pomacea Canaliculata and Screening of Target‐Based Molluscicidal Compounds.” Pesticide Biochemistry and Physiology 192: 105424. 37105626 10.1016/j.pestbp.2023.105424

[ece371581-bib-0038] Manara, E. , V. Cambi , and P. R. Martín . 2022. “Evaluating the Combined Use of Feeding Trials and a Micrographic Technique to Study the Natural Diet of *Pomacea canaliculata* .” Limnologica 97: 126022.

[ece371581-bib-0039] Martemyanov, V. I. , N. A. Berezina , A. S. Mavrin , and A. N. Sharov . 2021. “Shifted Mineral Ions Transport in the Mollusk *Unio pictorum* Exposed to Environmental Concentrations of Diclofenac.” Comparative Biochemistry and Physiology, Part C: Toxicology & Pharmacology 248: 109107. 34126253 10.1016/j.cbpc.2021.109107

[ece371581-bib-0040] Martyniuk, V. , V. Khoma , T. Matskiv , et al. 2022. “Indication of the Impact of Environmental Stress on the Responses of the Bivalve Mollusk *Unio tumidus* to Ibuprofen and Microplastics Based on Biomarkers of Reductive Stress and Apoptosis.” Comparative Biochemistry and Physiology. C 261: 109425. 10.1016/j.cbpc.2022.109425 35914710

[ece371581-bib-0041] Musri Musman, S. K. , S. Karina , and R. Rizqi . 2013. “Fina Arisca, Preliminary Study on the Anti Hatching of Freshwater Golden Apple Snail *Pomacea canaliculata* (Gastropoda: Ampullariidae) Eggs From *Barringtonia racemosa* (Magnoliopsida: Lecythidaceae) Seeds Extract.” AACL Bioflux 6, no. 4: 394–398.

[ece371581-bib-0042] Pan, J. , M. Shi , L. Li , et al. 2019. “Pterostilbene, a Bioactive Component of Blueberries, Alleviates Renal Fibrosis in a Severe Mouse Model of Hyperuricemic Nephropathy.” Biomedicine & Pharmacotherapy 109: 1802–1808. 30551434 10.1016/j.biopha.2018.11.022

[ece371581-bib-0043] Pan, X.‐D. , and J.‐L. Han . 2023. “Heavy Metals Accumulation in Bivalve Mollusks Collected From Coastal Areas of Southeast China.” Marine Pollution Bulletin 189: 114808. 36907167 10.1016/j.marpolbul.2023.114808

[ece371581-bib-0044] Planells, B. , I. Gómez‐Redondo , E. Pericuesta , P. Lonergan , and A. Gutiérrez‐Adán . 2019. “Differential Isoform Expression and Alternative Splicing in Sex Determination in Mice.” BMC Genomics 20, no. 1: 202. 30871468 10.1186/s12864-019-5572-x PMC6419433

[ece371581-bib-0045] Polyak, Y. M. , N. A. Berezina , D. E. Polev , and A. N. Sharov . 2022. “The State of the Intestinal Bacterial Community in Mollusks for Assessing Habitat Pollution in the Gulf of Finland (Baltic Sea).” Estuarine, Coastal and Shelf Science 278: 8095.

[ece371581-bib-0046] Price, J. , R. Taylor , S. Ramsay , G. Kettles , and P. Dominy . 2007. “Mutation of the AtNOS1/AtNOR1 Gene Confers Salt Tolerance on Arabidopsis.” Comparative Biochemistry and Physiology a—Molecular & Integrative Physiology 146, no. 4: S259–S260.

[ece371581-bib-0047] Purwaningsih, S. . 2015. “Effect of Boiling and Steaming on the Profile Fatty Acids and Cholesterol in Muscle Tissue of Molluscs.” International Food Research Journal 22, no. 3: 1087–1094.

[ece371581-bib-0048] Qin, Z. , M. Yang , J. E. Zhang , and Z. Deng . 2022. “Enhanced Salinity Tolerance of *Pomacea canaliculata* Through Acclimation to Lower Salinities.” Hydrobiologia 849, no. 13: 3015–3029.

[ece371581-bib-0049] Qin, Z. , M. Yang , J.‐E. Zhang , and Z. Deng . 2020. “Effects of Salinity on Survival, Growth and Reproduction of the Invasive Aquatic Snail *Pomacea canaliculata* (Gastropoda: Ampullariidae).” Hydrobiologia 847, no. 14: 3103–3114.

[ece371581-bib-0050] Quigley, D. A. , E. Kandyba , P. Huang , et al. 2016. “Gene Expression Architecture of Mouse Dorsal and Tail Skin Reveals Functional Differences in Inflammation and Cancer.” Cell Reports 16, no. 4: 1153–1165. 27425619 10.1016/j.celrep.2016.06.061 PMC5087975

[ece371581-bib-0051] Ren, Z. M. . 2020. “Transcriptome Analysis of the *Sepia pharaonis*: Identification of Low Salinity Stress‐Related Information and Microsatellite Markers.” Comparative Biochemistry and Physiology. Part D, Genomics & Proteomics 35: 705. 10.1016/j.cbd.2020.100705 32623150

[ece371581-bib-0052] Sakalauskaite, J. , F. Marin , B. Pergolizzi , and B. Demarchi . 2020. “Shell Palaeoproteomics: First Application of Peptide Mass Fingerprinting for the Rapid Identification of Mollusc Shells in Archaeology.” Journal of Proteomics 227: 103920. 32712371 10.1016/j.jprot.2020.103920

[ece371581-bib-0053] Salleh, N. H. M. , and D. Arbain . 2015. “Application of Crude Protease From Cheap and Local Raw Material as a Biopesticide for the Disruption of *Pomacea Canaliculata* Eggs.” International Journal of Environmental Science and Development 6, no. 4: 275–278.

[ece371581-bib-0054] Serba, D. D. , S. R. Uppalapati , N. Krom , et al. 2016. “Transcriptome Analysis in Switchgrass Discloses Ecotype Difference in Photosynthetic Efficiency.” BMC Genomics 17, no. 1: 1040. 27986076 10.1186/s12864-016-3377-8 PMC5162099

[ece371581-bib-0055] Sharma, V. , A. Nandan , H. Singh , et al. 2019. “Events of Alternative Splicing in Head and Neck Cancer via RNA Sequencing—An Update.” BMC Genomics 20, no. 1: 442. 31159745 10.1186/s12864-019-5794-y PMC6545735

[ece371581-bib-0056] Si, Y. , H. Wen , Y. Li , et al. 2018. “Liver Transcriptome Analysis Reveals Extensive Transcriptional Plasticity During Acclimation to Low Salinity in *Cynoglossus semilaevis* .” BMC Genomics 19, no. 1: 464. 29914359 10.1186/s12864-018-4825-4 PMC6006554

[ece371581-bib-0057] Slattery, M. L. , L. E. Mullany , L. C. Sakoda , R. K. Wolff , W. S. Samowitz , and J. S. Herrick . 2018. “Dysregulated Genes and miRNAs in the Apoptosis Pathway in Colorectal Cancer Patients.” Apoptosis 23, no. 3–4: 237–250. 29516317 10.1007/s10495-018-1451-1 PMC5856858

[ece371581-bib-0058] Swinehart, J. H. , A. P. Giannini , D. A. Rosenbaum , and M. A. Cheney . 1998. “Effects of Divalent Cations on Amino Acid and Divalent Cation Losses From and Glycine Influx Into Gills of Freshwater Bivalve Molluscs Anodonta Californiensis and *Corbicula manilensis* .” Colloids and Surfaces A: Physicochemical and Engineering Aspects 144, no. 1: 19–25.

[ece371581-bib-0059] Thakur, S. , S. Singh , and B. Pal . 2021. “Superior Adsorption Removal of Dye and High Catalytic Activity for Transesterification Reaction Displayed by Crystalline CaO Nanocubes Extracted From Mollusc Shells.” Fuel Processing Technology 213: 106707.

[ece371581-bib-0060] Vokhshoori, N. L. , T. C. Rick , T. J. Braje , and M. D. McCarthy . 2023. “Preservation of Stable Isotope Signatures of Amino Acids in Diagenetically Altered Middle to Late Holocene Archaeological Mollusc Shells.” Geochimica et Cosmochimica Acta 352: 36–50.

[ece371581-bib-0061] Wang, M. , Z. Chen , H. Zhang , H. Chen , and X. Gao . 2018. “Transcriptome Analysis Provides Insight Into the Molecular Mechanisms Underlying Gametophyte Factor 2‐Mediated Cross‐Incompatibility in Maize.” International Journal of Molecular Sciences 19, no. 6: 1757. 29899298 10.3390/ijms19061757 PMC6032218

[ece371581-bib-0062] Wang, S. , L. Zheng , M. Shen , et al. 2023. “Habitual Feeding Patterns Impact Polystyrene Microplastic Abundance and Potential Toxicity in Edible Benthic Mollusks.” Science of the Total Environment 866: 161341. 36603620 10.1016/j.scitotenv.2022.161341

[ece371581-bib-0063] Wang, X. , A. Ma , Z. Huang , Z. Sun , and Z. Liu . 2022. “Genetic Mechanism for Antioxidant Activity of Endogenous Enzymes Under Salinity and Temperature Stress in Turbot ( *Scophthalmus maximus* ).” Antioxidants (Basel) 11, no. 10: 2062. 36290784 10.3390/antiox11102062 PMC9598745

[ece371581-bib-0064] Wang, Z. , J. Tan , L. Tan , J. Liu , and L. Zhong . 2012. “Control the Egg Hatchling Process of *Pomacea canaliculata* (Lamarck) by Water Spraying and Submersion.” Acta Ecologica Sinica 32, no. 4: 184–188.

[ece371581-bib-0065] Xiong, Y. , L. Hu , Z. Yan , J. Zhang , and H. Li . 2018. “Transcriptomic Analysis of Embryo Development in the Invasive Snail *Pomacea canaliculata* .” Journal of Molluscan Studies 84, no. 3: 233–239.

[ece371581-bib-0066] Xu, P. , T. T. Wang , X. Z. Liu , et al. 2019. “Sirt6 Regulates Efficiency of Mouse Somatic Reprogramming and Maintenance of Pluripotency.” Stem Cell Research & Therapy 10, no. 1: 9. 30630525 10.1186/s13287-018-1109-5 PMC6329104

[ece371581-bib-0067] Yang, C. , Y. Huang , Z. Lu , et al. 2023. “Sublethal Effects of Niclosamide on the Aquatic Snail *Pomacea canaliculata* .” Ecotoxicology and Environmental Safety 259: 115064. 37229873 10.1016/j.ecoenv.2023.115064

[ece371581-bib-0068] Yang, S. , Q. Liu , Y. Wang , et al. 2016. “Effects of Dietary Supplementation of Golden Apple Snail (*Pomacea canaliculata*) Egg on Survival, Pigmentation and Antioxidant Activity of Blood Parrot.” Springerplus 1556, no. 5: 1–11. 10.1186/s40064-016-3051-2 PMC5021657 27652129

[ece371581-bib-0069] Yang, S. , J.‐r. Zhao , L.‐l. Wu , et al. 2018. “The Salinity Tolerance of the Invasive Golden Apple Snail (*Pomacea canaliculata*).” Molluscan Research 38, no. 2: 90–98.

[ece371581-bib-0070] Zhang, N. , H. Yu , H. Yu , et al. 2018. “A Core Regulatory Pathway Controlling Rice Tiller Angle Mediated by the LAZY1‐Dependent Asymmetric Distribution of Auxin.” Plant Cell 30, no. 7: 1461–1475. 29915152 10.1105/tpc.18.00063 PMC6096585

[ece371581-bib-0071] Zhang, X. , H. Wen , H. Wang , Y. Ren , J. Zhao , and Y. Li . 2017. “RNA‐Seq Analysis of Salinity Stress‐Responsive Transcriptome in the Liver of Spotted Sea Bass (Lateolabrax Maculatus).” PLoS One 12, no. 3: e0173238. 28253338 10.1371/journal.pone.0173238 PMC5333887

[ece371581-bib-0072] Zhou, K. , Y. Huang , Z. Chen , et al. 2020. “Liver and Spleen Transcriptome Reveals That *Oreochromis aureus* Under Long‐Term Salinity Stress May Cause Excessive Energy Consumption and Immune Response.” Fish & Shellfish Immunology 107: 469–479. 33181338 10.1016/j.fsi.2020.11.010

[ece371581-bib-0073] Zhou, X. M. , X. Zhou , Y. Chen , et al. 2016. “The Complete Mitochondrial Genome of *Pomacea canaliculata* (Gastropoda: Ampullariidae).” Mitochondrial DNA Part A DNA Mapping, Sequencing, and Analysis 27, no. 2: 884–885. 24865920 10.3109/19401736.2014.919488

