# Correction to “Integrated Analysis of Survival, Physiological‐Biochemical, and Transcriptomic Changes Reveals the Impact of Saline Stress on the Freshwater Snail *Pomacea canaliculata*”

**DOI:** 10.1002/ece3.71833

**Published:** 2025-07-30

Chen, Y., Yao, F., Shi, Z., et al. 2025. “Integrated Analysis of Survival, Physiological‐Biochemical, and Transcriptomic Changes Reveals the Impact of Saline Stress on the Freshwater Snail *Pomacea canaliculata*.” *Ecology and Evolution* 15: e71581. https://doi.org/10.1002/ece3.71581.

In **Figure 1** “Morphological indicators of *P. canaliculata*,” there were some omission errors involving four indicators: height from all layers (HL), inner lip length (IL), basal lip height (BL), and aperture inner length (AL'), their measurement protocols or standards were not labelled and indicated in the figure. These errors should be corrected to the new Figure 1 provided below, which includes all measured morphological indicators.

**FIGURE 1:**
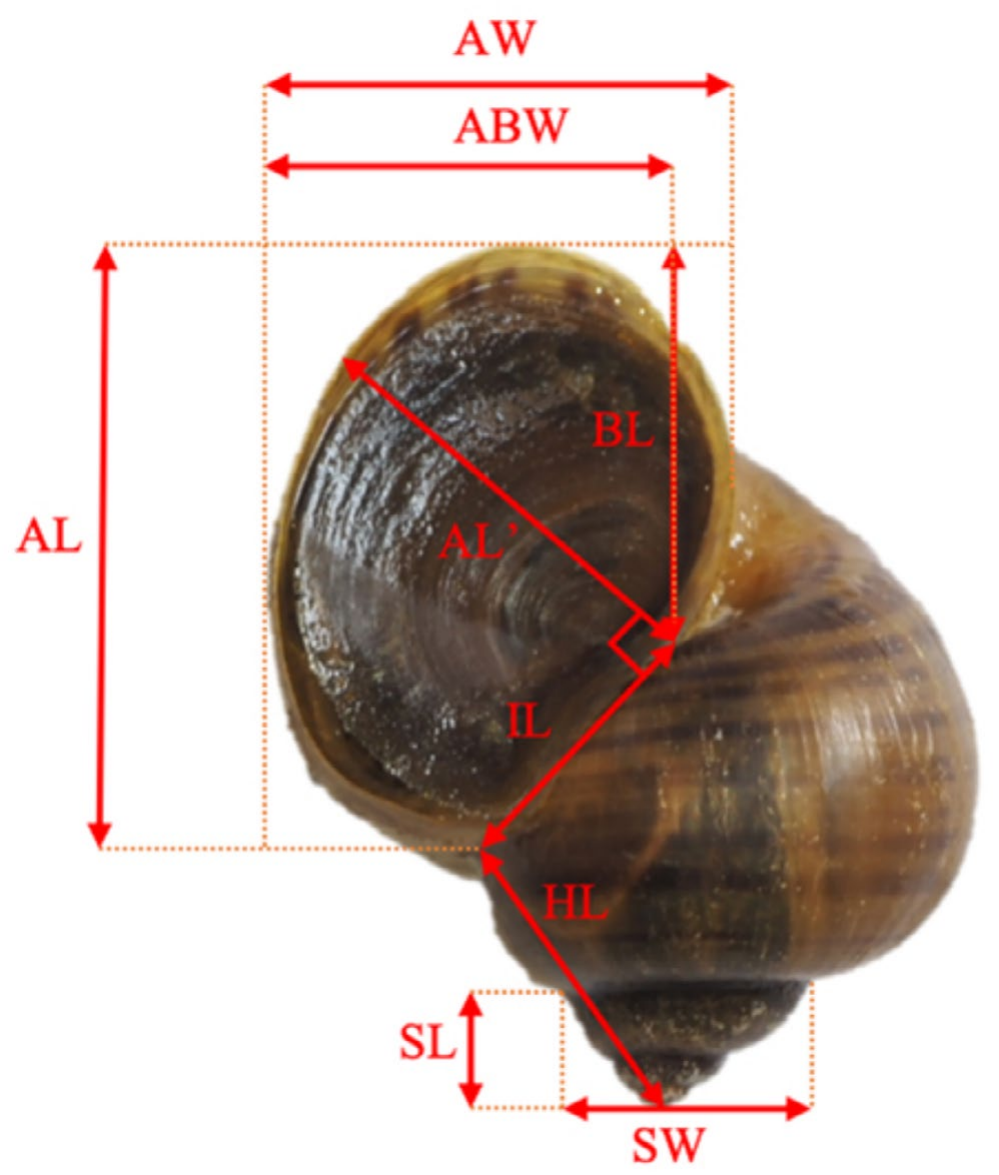
Morphological indicators of *P. canaliculata*.

In **Figure 13** “Schematic diagrams showing changes in physiology of *P. canaliculata* under saline stress treatments, based on the results obtained,” the effects of saline stress treatment on “Lip Content,” “Glycerol Content,” and “Cholesterol Content” were omitted, including indications of whether they were promoted or reduced (shown with arrows and “+” or “–” symbols). These errors should be corrected to the new Figure 13 provided below.

**FIGURE 13:**
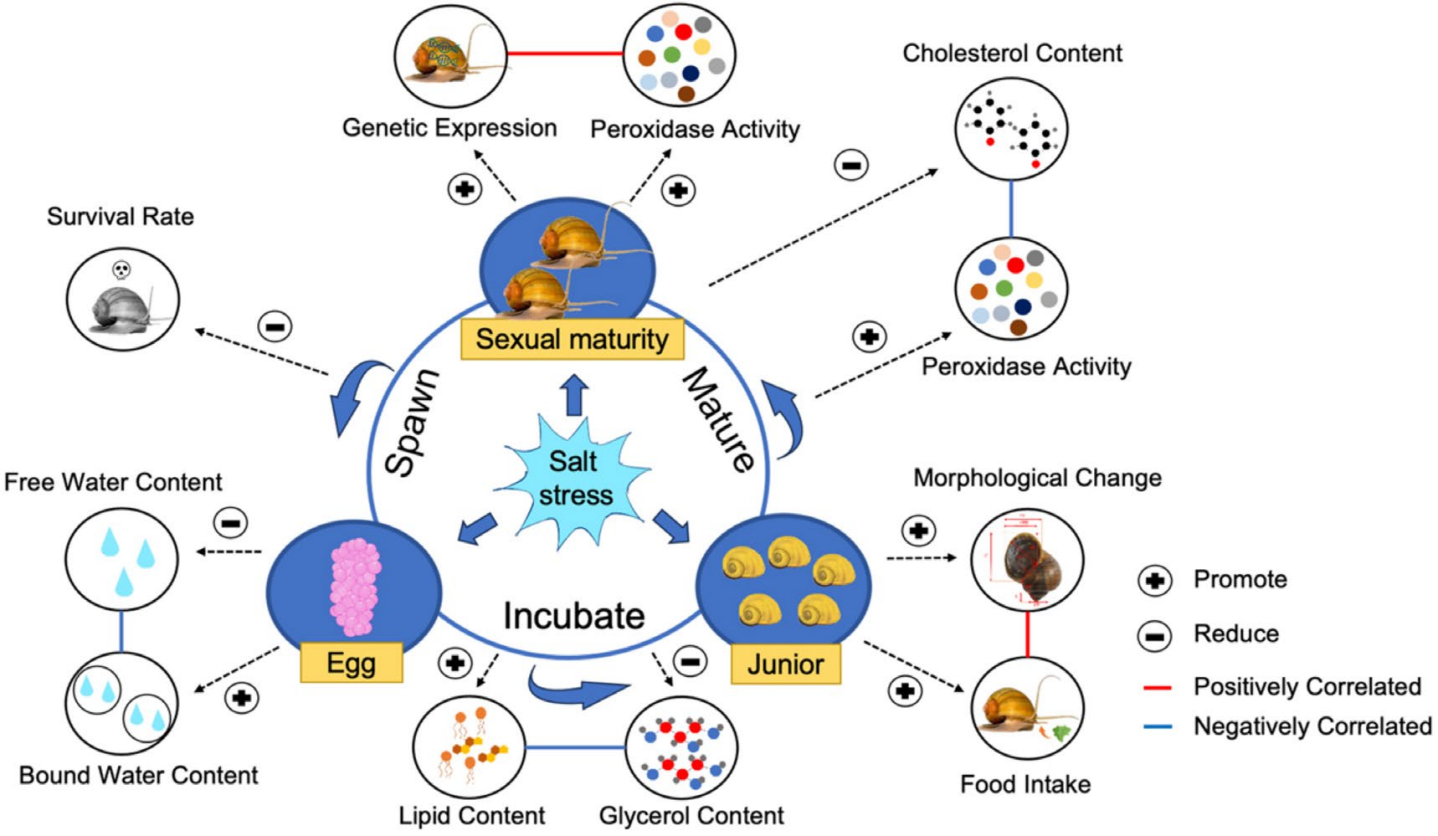
Schematic diagrams showing changes in the physiology of *P. canaliculata* under saline stress treatments, based on the results obtained.

We apologize for this error.

